# Toward simple, rapid, and deep plant proteome analysis with an in-cell proteomics strategy

**DOI:** 10.1093/plphys/kiag557

**Published:** 2026-07-30

**Authors:** Deji Adekanye, Jasmine Parks, Meghana Kusuru, Jeffrey L Caplan, Yanbao Yu

**Affiliations:** Department of Biological Sciences, University of Delaware, Newark, DE 19716, United States; Department of Chemistry and Biochemistry, University of Delaware, Newark, DE 19716, United States; Department of Computer and Information Sciences, University of Delaware, Newark, DE 19716, United States; Department of Biological Sciences, University of Delaware, Newark, DE 19716, United States; Department of Plant and Soil Sciences, University of Delaware, Newark, DE 19716, United States; Department of Chemistry and Biochemistry, University of Delaware, Newark, DE 19716, United States

## Abstract

While liquid chromatography-mass spectrometry (LCMS) has revolutionized plant proteomics over the past decade, plant sample preparation remains a major challenge due to rigid cell walls, abundant secondary metabolites, and wide dynamic range of protein abundance. These hurdles demand laborious tissue disruption, complex precipitation, and extensive cleanup prior to LCMS analysis, limiting the widespread adoption of proteomic technologies within the plant biology community. To overcome these barriers, we introduced an “in-cell proteomics” strategy that bypasses cell lysis and protein extraction by performing digestion directly inside methanol-fixed cells. We systematically benchmarked this strategy against conventional lysate-based workflows across 4 model plants (*Arabidopsis thaliana*, *Nicotiana benthamiana*, *Zea mays*, and *Sorghum bicolor*) and 3 tissue types (leaves, pollen, and seeds). Combined with minimal input material and single-shot LCMS, the in-cell approach consistently identified 9,000 to 12,000 proteins from leaves, 7,000 to 9,000 from pollen grains, and approximately 8,000 from seeds. Our comprehensive dataset demonstrates that this in-cell digestion approach substantially simplifies plant sample preparation while delivering proteomic performance equivalent to established workflows. Finally, to demonstrate the biological utility of this approach, we characterized the proteomes of *N. benthamiana* leaves infected with 2 fungal strains that exhibit different host specificities. Our in-depth proteomic data revealed distinct host response signatures differentiating the host-adapted *Colletotrichum destructivum* from the nonhost-adapted *Colletotrichum sublineola* strain. Overall, this study provides a simple, unbiased alternative for plant proteomic analysis that can be readily applied to tackle complex agricultural and physiological challenges in plant biology.

## Introduction

Plants are fundamental to both food and human health, providing essential nutrients, supporting ecosystems, and contributing to overall well-being (Raskin et al. 2002; Martin et al. 2011). To advance our understanding of plant biology, liquid chromatography and mass spectrometry (LCMS)-based proteomics technologies have been widely adopted in recent years, playing an increasingly important role in elucidating protein functions, interactions, and responses to environmental stimuli (Mergner and Kuster 2022; Geddes-McAlister and Uhrig 2025). In conventional bottom-up proteomics, one of the most critical steps is tissue disruption and protein extraction. Compared to cultured cells, protein extraction from plant tissues is particularly challenging because plant cells possess rigid cell walls, a low protein-to-volume ratio, and a diverse array of metabolites that can interfere with enzymatic digestion and downstream LC-MS/MS analyses. Consequently, efficient sample preparation often requires extensive mechanical disruption, protein cleanup procedures, and relatively large amounts of starting material (Isaacson et al. 2006; Mergner and Kuster 2022). Recent advances in sample preparation technologies have helped address these challenges through the adaptation of modern filter- or bead-based workflows, including filter-aided sample preparation (FASP) (Szymanski et al. 2017; Song et al. 2018), protein suspension trapping (S-Trap) (Amnan et al. 2022; Chen et al. 2023), and single-pot solid-phase-enhanced sample preparation (SP3) (Mikulášek et al. 2021; Brajkovic et al. 2023). These approaches enable the processing of protein lysates containing strong detergents or high concentrations of chaotropic agents, conditions that can facilitate protein extraction and solubilization (Mergner and Kuster 2022). As a result, these approaches have become widely adopted across the plant proteomics community. Furthermore, a number of studies have compared different sample preparation workflows to assess their performance in terms of proteome coverage, quantitative accuracy, reproducibility, and cost-effectiveness across diverse plant systems, which have been extensively reviewed elsewhere (Bahmani et al. 2021; Balotf et al. 2022).

In addition to protein sample processing, several depletion and predigestion fractionation strategies have been developed to remove highly abundant proteins, such as ribulose-1,5-bisphosphate carboxylase/oxygenase (RuBisCO), thereby enhancing the detection of low-abundance proteins (Gupta et al. 2015; Tola and Missihoun 2023). Post-digestion fractionation approaches, including ion-exchange LC, have also been widely employed to reduce sample complexity and improve proteome coverage prior to LCMS analysis (Song et al. 2018; Zhang et al. 2019; Mergner et al. 2020). To further balance analytical depth with sample throughput, several advanced MS-based technologies have been adopted in plant proteomics. These include high-field asymmetric waveform ion mobility spectrometry (FAIMS), sequential window acquisition of all theoretical mass spectra (SWATH-MS), and other data-independent acquisition (DIA) strategies. Such approaches enhance proteomics sensitivity and proteome coverage by reducing chemical noise, filtering out interfering ions (eg, singly charged species), and improving the consistency and comprehensiveness of peptide detection and quantification (Zhang et al. 2019; Mu et al. 2022; Rodriguez Gallo et al. 2023).

Recently, an on-filter in-cell (OFIC) proteomics strategy has emerged as a promising alternative to conventional lysate-based proteomics sample preparation workflows (Martin et al. 2024). Unlike traditional approaches, OFIC eliminates the need for cell lysis and protein extraction by digesting proteins directly within intact cells following a simple methanol-based fixation step. In this process, methanol serves both as a permeabilizing agent and a fixative by dissolving membrane lipids, dehydrating cells, and precipitating proteins. These effects increase membrane permeability, enabling proteolytic enzymes to access intracellular proteins and subsequently digest the denatured proteome. A notable advantage of the OFIC workflow is that it integrates multiple sample preparation steps within a single filter device. In addition to protein digestion, the method incorporates protein pretreatment steps, including reduction, alkylation, and post-digestion peptide desalting (Martin et al. 2024). By consolidating the entire sample preparation workflow into a single platform, OFIC substantially simplifies sample handling, minimizes sample loss, and reduces the amount of starting material required for proteomic analysis. To date, the OFIC strategy has been successfully applied to a diverse range of biological samples, including *Xenopus* embryos (Xu et al. 2025; Sun et al. 2026), cultured mammalian cells such as neuroblastoma cells (Moafian et al. 2025), bacteria (Keffer et al. 2025; Zhou et al. 2025), and other difficult-to-lyse organisms such as yeast and *Caenorhabditis elegans* worms (Elsayyid et al. 2025; Nwaora et al. 2026). However, plant tissues present a distinct dimension of challenges compared to these model systems. The presence of rigid cell walls, abundant secondary metabolites, and highly heterogeneous tissue structures can complicate direct in-cell proteolysis. Consequently, whether the OFIC approach can be successfully extended to plant proteomics remains an open question and warrants systematic investigation.

In this study, we evaluated the applicability of the OFIC proteomics workflow for the first time to plant tissues using *Sorghum bicolor* (hereafter *Sorghum*) leaves as a proof-of-concept system. Through comprehensive, head-to-head comparisons with conventional detergent- and acid-based lysis methods, we demonstrated that plant leaf tissues can be effectively processed using the OFIC strategy for in-depth proteome analysis. We further extended the application of OFIC to 3 additional model plant species, *Arabidopsis thaliana* (hereafter *A. thaliana*), *Zea mays* (hereafter *maize*), and *Nicotiana benthamiana* (hereafter *N. benthamiana*), as well as 2 additional plant tissue types, pollen and seeds. Across all sample types examined, comparisons with corresponding lysate-based digestion workflows consistently demonstrated the robustness and versatility of the in-cell approach, establishing OFIC as a powerful and broadly applicable platform for plant proteomics. Last, we applied the OFIC approach to address a biologically significant question: how the proteome of *N. benthamiana* leaves dynamically changes upon inoculation with host-adapted *Colletotrichum destructivum* and nonhost-adapted *Colletotrichum sublineola* fungi. With in-cell proteomics, we were able to gain insights into how adapted *C. destructivum* suppresses or induces different *N. benthamiana* defense responses.

## Materials and methods

### Plant growth, fungal treatment, and sample collection

Transgenic *N. benthamiana* plants expressing the N nucleotide-binding leucine-rich repeat (NLR) immune receptor were grown in a light chamber at 150 μmol m^−2^ s^−1^ light intensity at 23 °C for 3 to 6 weeks until the 8-leaf stage, with a diurnal cycle of 18 hours light and 6 hours dark. *Zea mays* (B73) and *Sorghum bicolor* (BTX 623) seeds were grown for 14 days in a 6-inch pot containing Pro-Mix BK55 growing medium in a greenhouse or growth chamber with a16/8 hour light/dark cycle at a temperature of 27 °C and a light intensity of 350 μmol m^−2^ s^−1^, maintaining 65% relative humidity. *A. thaliana* (Col-0) plants were grown at 100 μmol m^−2^ s^−1^ light intensity at 20 °C for 2 to 3 weeks, with a diurnal cycle of 16 hours light and 8 hours dark.

Cultures of *C. sublineola* and *C. destructivum* were prepared from stocks of their respective spores and stored in 15% glycerol and 1×potato dextrose broth at −80 °C. Spore stocks were grown on Mathur's media (2.8 g/L glucose, 2.2 g Mycological peptone, 1.2 g/L MgSO_4_×7H_2_O, 2.7 g/L KH_2_PO_4_, and 30 g/L agar, pH 5.5) at 28 °C under continuous fluorescent lighting, and conidia were harvested after 7 to 12 d. Droplets of conidia diluted to 2 × 106 conidia ml^−1^ were applied to *N. benthamiana* leaves using a pipette and maintained under humid condition at 28 °C. After 96 h, *N. benthamiana* leaf samples were collected and immediately stored on ice for protein extraction.

### Multiphoton microscopy and image collection

Approximately 1 mm × 1 mm leaf sections were treated with methanol or the water control for 5 min and then stained with the cell wall dye Calcofluor White (CW) for 1 min. Multiphoton microscopy was conducted on a Zeiss LSM880 NLO microscope (Carl Zeiss, Inc, Dublin, CA, United States) equipped with a Chameleon Discovery NX (Coherent, Inc., Santa Clara, CA, United States) multiphoton laser tuned to 745 nm at 2% power. Fluorescence emission was collected with a band pass (BP) of 410 to 481 nm for CW. A z-stack with a *z*-interval size of 0.310 μm was acquired and represented in Fiji/ImageJ (Schindelin et al. 2012) as a maximum intensity projection or cross-sectional views. To document chlorophyll extraction, 1 mm × 1 mm leaf sections were treated with methanol for 0, 5, 15, or 30 min, and then color images were acquired on an Axio Observer 7 AI (Carl Zeiss, Inc, Dublin, CA, United States).

### Sample processing for proteomics with on-ilter in-cell, sodium dodecyl sulfate, and trifluoroacetic acid

For the OFIC-based sample preparation, E3filters (CDS Analytical, Oxford, PA, United States) were used following an in-cell digestion protocol described previously (Martin et al. 2024). A detailed step-by-step protocol is provided in Supplementary Protocol 1. In brief, the leaf tissues derived from different plants were first sliced into 1 to 2 cm^2^ sections, further cut into 1 to 2 mm^2^ small pieces and then transferred to E3filters. The cutting and slicing should be performed promptly on ice or under low temperature. Alternatively, the leaf tissue may be ground in liquid nitrogen, for instance, using the pellet pestle with a cordless motor (Fisher Scientific; catalog No. 12-141-361) before transferring to the filters. For *sorghum* and *maize* leaf, around 2-cm proximal leaf sections were sliced and treated as biological replicates; for *N. benthamiana* and *A. thaliana* leaf, around 2-cm^2^ leaf sections and a single rosette leaf were used as biological replicates, respectively, for each digestion experiment. For all sample types, at least 3 replicate experiments were performed for each digestion method throughout this study, unless otherwise stated. Afterward, the E3filters were vortexed with 400 μL of pure methanol at 1,000 rpm for 5 min, and then centrifuged at 3,000 rpm for 1 min to discard the flow through. The above vortexing and centrifugation steps were repeated 3 to 5 times until the green chlorophyll disappeared. The samples were then incubated with 10 mM tris (2-carboxyethyl) phosphine (TCEP) and 40 mM chloroacetamide (CAA) in 200 μL of 50 mM triethylammonium bicarbonate (TEAB) at 45°C for 10 min, followed by a quick spin and a wash step with 200 μL of 50 mM TEAB. Unless otherwise specified, the centrifugation in this study is 3,000 rpm for 1 to 2 min. Afterward, the samples were transferred to clean collection tubes and subjected to tryptic digestion in the presence of 200 μL of 50 mM TEAB and 1 μg of trypsin (Promega, WI). The samples were incubated at 37°C for 16 to 18 hours with gentle shaking (300 rpm). After digestion, the filters were spun to collect flow through followed by 2 sequential elution, 200 μL of 0.2% formic acid (FA) in water, and 200 μL of 50% acetonitrile (ACN) in water, respectively. The elution was pooled, dried in SpeedVac, and desalted using C18-based StageTips (CDS Analytical, Oxford, PA, United States). The dried peptides were stored under −80°C until further analysis.

For the sodium dodecyl sulfate (SDS)-based preparation, the sliced leaf pieces or ground leaf powder in liquid nitrogen were first lysed with 100 μL SDS buffer (4% SDS, 100 mM Tris-HCl, pH 8.0) by vortexing at 1,500 rpm for 10 min, and then sonicated in a water bath for another 10 min. The samples were then boiled with 10 mM TCEP and 40 mM CAA at 95°C for 10 min and cleared at 13,000 rpm for 15 min to collect the supernatant lysate. The protein lysates were processed following a standard E3 protocol reported recently (Martin et al. 2024). In brief, the lysates were mixed with 4× volume of 80% ACN to induce protein aggregation, then transferred to E3filters. Next, the filters were washed 2 to 3 times with 80% ACN, spun, and then the flow through was discarded. The samples were digested and desalted following the same procedure as described above. For trifluoroacetic acid (TFA)-based sample preparation, the leaf pieces were incubated with 100 μL of pure TFA at room temperature for 10 min and centrifuged at 13,000 rpm for 10 min to pellet debris. The supernatant lysates were mixed with 4× volume of pure acetone to induce protein aggregation and then transferred to E3filters. This step was followed by 1 wash with 200 μL of acetone and another wash with 200 μL of 50 mM TEAB. The samples were then mixed with 10 mM TCEP and 40 mM CAA in 200 μL of 50 mM TEAB, and incubated at 45 °C for 10 min. The E3filters were then washed once with 200 μL of 50 mM TEAB followed by the same digestion and peptide desalting procedures as described above.

For pollen sample preparation, a detailed step-by-step protocol is provided in Supplementary Protocol 2. Basically, to collect mature *N. benthamiana* and *A. thaliana* pollen grains, dehiscent anthers were first gently cut off the plant and then transferred to 1.5 mL low-binding tubes mixed with 0.5 mL of cold phosphate buffered saline, and then vortexed at 1,000 rpm for 5 min. The tube was centrifuged at 3,000 rpm to remove floral debris and then rinsed once again with 0.5 mL cold PBS. Due to limited availability, pollen grains from 2 *A. thaliana* flowers were pooled and subjected to the OFIC method processing. *N. benthamiana* pollen grains from 1 flower were collected, and split into 4 equal aliquots for both OFIC and SDS lysate-based digestion (ie, 2 replicates for each method). For the OFIC method, pollen aliquots were transferred to E4tips (CDS Analytical, Oxford, PA) that were prefilled with 200 μL of pure methanol, spun and discard flow through, mixed with 200 μL of pure methanol again, and incubated at 4°C for 10 to 15 min. The following procedures, including reduction and alkylation, digestion, and peptide desalting were performed following a similar in-cell digestion procedure reported recently (Elsayyid et al. 2025). In brief, after incubation, the E4tips were spun at 4,000 rpm for 2 to 3 min to discard the flow through, and then subjected to reduction and alkylation by adding final concentration of 10 mM TCEP and 40 mM CAA with 200 μL of 50 mM TEAB and incubating at 45 °C for 10 min. The tips were spun, washed once with 200 μL of 50 mM TEAB, and then transferred to new collection tubes. For digestion, 2.0 μg of trypsin and 150 μL of 50 mM TEAB buffer were added to the E4tips, which were then incubated at 37°C for 16 to 18 hours with gentle shaking. After digestion, about 10 μL of pure formic acid was added to the E4tips, which were then incubated under room temperature for 3 to 5 min. The tips were then centrifuged at 1,500 rpm for 10 min to allow the peptides to bind to the resins, followed by a wash step with 200 μL of 0.5% acetic acid in water. The E4tips were transferred to new collection tubes and eluted sequentially with 200 μL of 0.5% acetic acid in 60% ACN and another 200 μL of 0.5% acetic acid in 80% ACN. The elution was dried in SpeedVac and stored under −80°C until further analysis. For the SDS lysate experiments, the pollen aliquots were first spun to discard any liquid, then ground under liquid nitrogen, followed by boiling with 50 μL of SDS buffer (4% SDS, 100 mM Tris-HCl, pH8) at 95°C for 10 min, and further sonication in water bath for 5 min. Afterward, the samples were transferred to E3 tips (CDS Analytical, Oxford, PA) that were prefilled with 200 μL of 80% ACN, mixed gently and then spun to discard flow through. The following procedures, including washing, reduction and alkylation, digestion, and desalting were performed using stacked tips (ie, with C18 tip) following a similar protocol described recently (Moafian et al. 2025).

For seed sample preparation, the *A. thaliana* seeds from 1 pod (around 15 to 20 seeds per pod) were used as 1 biological replicate for the digestion experiments. The seeds were first transferred to microtubes and ground in liquid nitrogen. For the OFIC method, the fine powder was incubated with 200 μL of pure methanol on ice for 10 to 15 min, and then transferred to E3tips XL (CDS Analytical, Oxford, PA, United States) followed by chlorophyll depletion, reduction/alkylation, digestion, and peptide desalting, similar to the procedures described above. Detailed protocol can be found in Supplementary Protocol 3. For the SDS method, the seed powders were first mixed with 50 μL of SDS buffer (4% SDS, 100 mM Tris-HCl, pH8) and then boiled at 95°C for 10 min. The lysate samples were further sonicated in a water bath for 5 to 10 min. Afterward, the samples were transferred to E3 tips (CDS Analytical, Oxford, PA, United States) that were prefilled with 200 μL of 80% ACN, mixed gently, and then spun to discard flow through. The following procedures were performed following a similar protocol described recently (Moafian et al. 2025).

### LC-MS/MS

The LC-MS/MS analysis was performed using an UltiMate 3000 RSLCnano system that was coupled to an Orbitrap Eclipse mass spectrometer and FAIMS Pro Interface (Thermo Scientific). The trap column was PepMap100 C18 with dimensions of 300 μm × 2 mm, and a particle size of 5 μm (Thermo Scientific). The analytical column was PepMap100 C18 (50 cm × 75 μm i.d., 3 μm particle size; Thermo Scientific) running at a flow of 250 nL/min. A linear LC gradient was applied from 1% to 25% mobile phase B (0.1% formic acid in acetonitrile) over 125 min, followed by an increase to 32% mobile phase B over 10 min. The column was washed with 80% mobile phase B for 5 min, followed by equilibration with mobile phase A (0.1% formic acid in water) for 15 min. For MS analysis, the data-independent acquisition (DIA) mode was used. In brief, the detector type was Orbitrap with a resolution of 60,000 for full scan; Precursor MS range (m/z) was 380 to 980; AGC target was Standard; Maximum injection time mode was Auto. For DIA MS/MS analysis, Isolation mode was Quadrupole; DIA Window type was Auto and Isolation Window (m/z) was 8 with an overlap of 1 m/z; activation type was HCD with fixed collision energy mode (30%); Detector Type was Orbitrap with a resolution of 15,000; Normalized AGC target (%) was 800, and Maximum injection time mode was Auto; the Loop Control was 2 s. For FAIMS compensation voltage (CV) setting, a 3-CV combination (−40, −55, and −75) was applied.

### Data analysis

The MS raw data were processed using Spectronaut software (version 19.8) and a library-free DIA analysis workflow with directDIA + and the plant proteomes obtained from UniProt Knowledgebase (*A. thaliana*, taxonomy ID: 3702, 136,324 sequences; *Sorghum bicolor*, taxonomy ID: 4558, 82,122 sequences; *Zea mays*, taxonomy ID: 4577, 207,460 sequences). The *N. benthamiana* protein database was curated in-house, containing 53,411 sequences from the NbDE database (Kourelis et al. 2019) combined with the Nicomics database (Wang et al. 2024). Detailed settings in Spectronaut included: Trypsin/P as the specific enzyme; peptide length from 7 to 52 amino acids; allowing 2 missed cleavages; toggle N-terminal M turned on; Carbamidomethyl on C as fixed modification; Oxidation on M and Acetyl at protein N-terminus as variable modifications; False discovery rates (FDRs) at PSM, peptide and protein level all set to 0.01; Quantity MS level set to MS2, and cross-run normalization turned on. Bioinformatics analyses including *t*-test, correlation, volcano plot, and clustering analyses were performed using Perseus software (version 2.0.11.0) and GraphPad Prism (version 10.5) unless otherwise indicated. Gene Ontology analysis was conducted by using local BlastP to identify the closest *A. thaliana* orthologs, which were used as the input in GeneCodis4 for cellular component GO and biological process GO (Garcia-Moreno et al. 2022). The STRING protein network was built using the differentially expressed proteins between *C. destructivum* and *C. sublineola*-inoculated *N. benthamiana* leaves (FDR 0.05, S_0_ = 0.1; ≥2 unique peptides). The protein identifiers were converted to the accessions of *A. thaliana* homologs obtained using BLASTP, and then imported into Cytoscape (version 3.10.3) via the STRING app using the full STRING network configuration. Interactions with a confidence score ≥0.40 and classified as experimentally validated or curated were selected. The resulting interaction data were imported into Node degree, and clustering coefficient and betweenness centrality are computed as the network topological parameters to assess the protein connectivity. Node colors were coded according to fold changes (FC), and node sizes were weighted by *P* values. Functional modules were identified using the MCODE plugin and annotated based on biological function and pathway enrichment.

## Results and discussion

### Benchmarking in-cell proteomics analysis of *sorghum* leaf

We first investigated whether methanol treatment could render plant leaf tissues accessible to proteolytic digestion. Brightfield imaging data suggested that, upon methanol fixation, *sorghum* leaf sections showed a progressive clearing of chlorophyll over 30 min (Figure S1a). To examine the cellular details, we labeled the cell wall with Calcofluor White (CW) dye and conducted multiphoton microscopy (Figure S1b). Methanol clearing resulted in improved, deeper staining and imaging of the cell wall of *sorghum* leaf sections. The tissue appeared to be more fragile after methanol treatment, and surface damage from handling the sample during the imaging were observed. Interestingly, cracks in the cell wall were also observed at the junctions of *sorghum* cells (Figure S1b). The changes in the cell wall may make the cell wall more porous and penetrable for proteolytic enzymes, since we recently showed that methanol fixation facilitated penetration of trypsin through the thick cuticles of *C. elegans*, and enabled direct digestion of proteins inside the intact worms (Elsayyid et al. 2025). We next evaluated whether repeated methanol exchanges further improved tissue clearing. Vortexing with fresh methanol every five minutes for 30 min (six exchanges) completely removed chlorophyll signal (Figure S1c), thereby minimizing potential interference from chlorophyll and other primary and secondary metabolites during LCMS analysis. This improved sample cleanliness is expected to enhance detection of low-abundance proteins.

**Figure 1:**
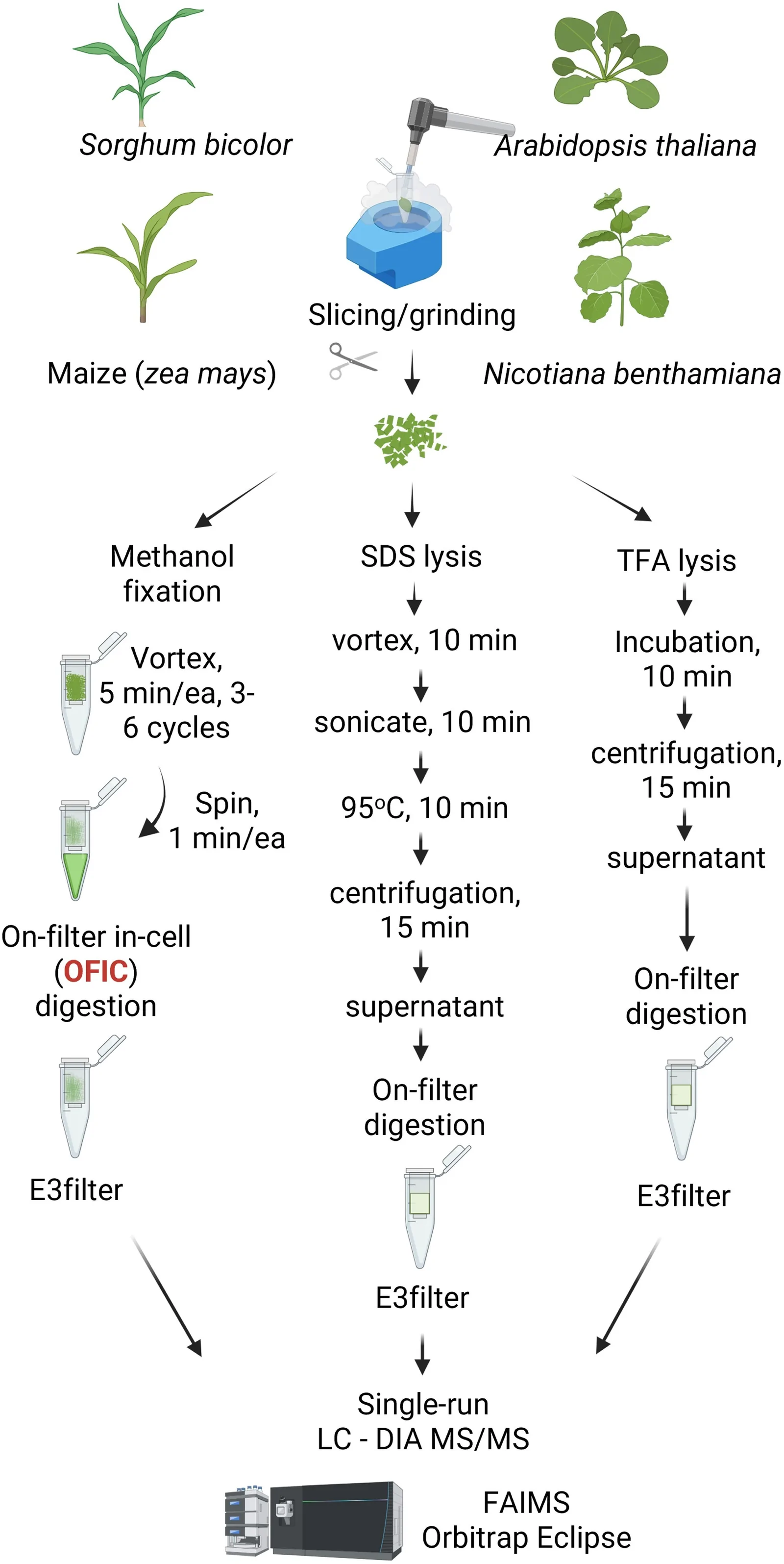
Illustration of plant proteomics workflows investigated in this study. Plant leaf tissues were first sliced or ground into small sections, and were then subjected to methanol-based chlorophyll depletion and fixation, and on-filter in-cell digestion. In parallel, leaf sections were lysed with SDS-based buffer or strong acid TFA followed by on-filter digestion. A single-shot LCMS analysis was performed using Orbitrap Eclipse with FAIMS interface. The flowchart was created by Biorender.

**Figure 2:**
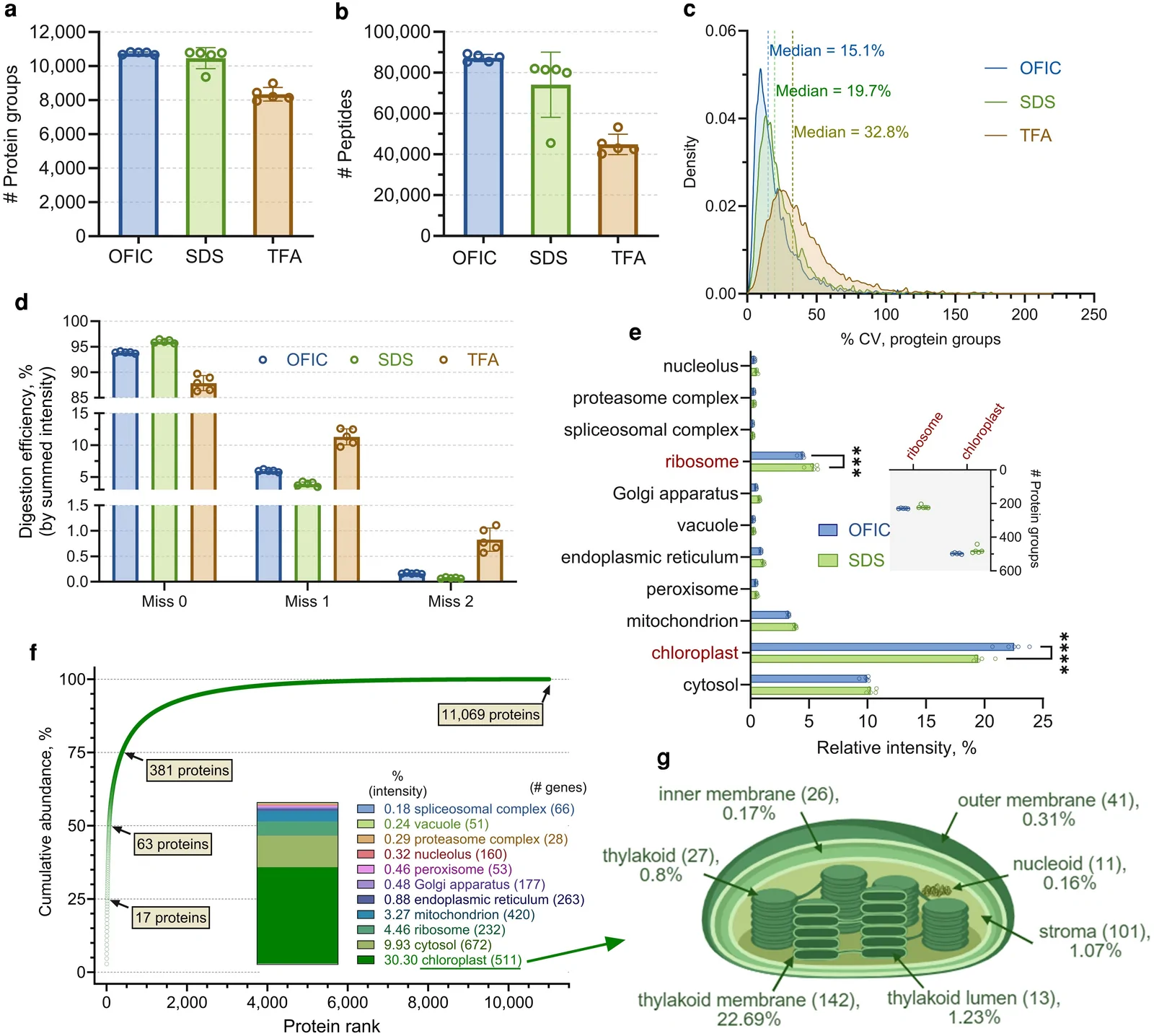
Benchmarking in-cell proteomics analysis of sorghum leaf. a–b Number of protein groups and peptides identified by the 3 processing methods, respectively. Error bars represent five biological replicates. c) Profiles of protein groups coefficient of variation. d) Digestion efficiency assessment based on peptide intensity. e) Cellular compartment analysis of the proteins derived from OFIC and SDS-based processing method. f) Cumulative protein abundance analysis of sorghum proteome derived from OFIC processing. The numbers of proteins account for 25%, 50%, and 75% of total protein mass are indicated in the plot. Inner diagram shows relative abundance of the eleven protein categories. g) Histogram shows the relative abundance of chloroplast-associated proteins. The number of proteins for each category was indicated in the brackets. Created with BioRender.com.

Physical disruption, such as grinding or blending frozen tissue followed by protein extraction with SDS buffer, has been commonly used for plant leaf proteomics (Mikulášek et al. 2021; Balotf et al. 2022). On the other hand, TFA was shown very recently to be able to dissolve hard-to-lyse cells such as gram-positive bacteria and microbiome samples (Doellinger et al. 2020). To our knowledge, TFA has not been applied to plant leaf proteomics. In this study, we thoroughly compared OFIC processing with SDS- and TFA-lysate based digestion methods. We took five proximal leaves derived from 4 to 5-week-old *sorghum* plants, and sliced them into approximately 2 × 2 cm sections. We treated each section as 1 biological replicate, and further cut it into 1 to 2 mm small pieces, which were then transferred to E3filters followed by methanol fixation, reduction and alkylation, protein digestion, and single-shot LCMS analysis (Supplementary Protocol I). The cutting and slicing here did not induce proteome-wide alterations in comparison to typical cryogenic grinding, which will be described later. The SDS- and TFA-based digestion experiments were performed in parallel (Fig. 1). Our data indicated that after a simple methanol fixation, surprisingly, the leaf slices were readily accessible for digestion, as evidenced by over 10,000 protein groups and nearly 90,000 peptide identifications from as little as ¼ of a single *sorghum* leaf (Fig. 2a–b; Table S1). Less than 1% of the proteins were exclusively identified by the OFIC method; the vast majority of them were also identified by at least 1 of the lysate-based digestion methods (Figure S2a), suggesting that in-cell processing is not prone to proteome-wide bias or under-representation. Because the OFIC method drastically streamlined proteomics sample preparation, we observed much reduced processing time (ie, < 20 min of centrifugation time compared to over 200 min by FASP) (Song et al. 2018), minimal sample requirement, higher reproducibility and quantitative accuracy compared to the lysate-based processing methods, which is consistent with what we observed from the worm experiments (Elsayyid et al. 2025). The coefficient of variance (CV) of protein groups was around 15% and the Pearson's correlation was near 0.97 between replicate experiments, both of which were better than SDS- and TFA-lysate-based methods (Fig. 2c; Figure S2b). Even though the proteins remained inside the mostly intact leaf tissue, the enzymatic digestion was minimally affected. Less than 18% of the total number of detected peptides carried miscleaved sites, and their abundance was low, accounting for only 6% to 7% of the total peptide intensity (Fig. 2d; Figure S2c).

**Figure 3:**
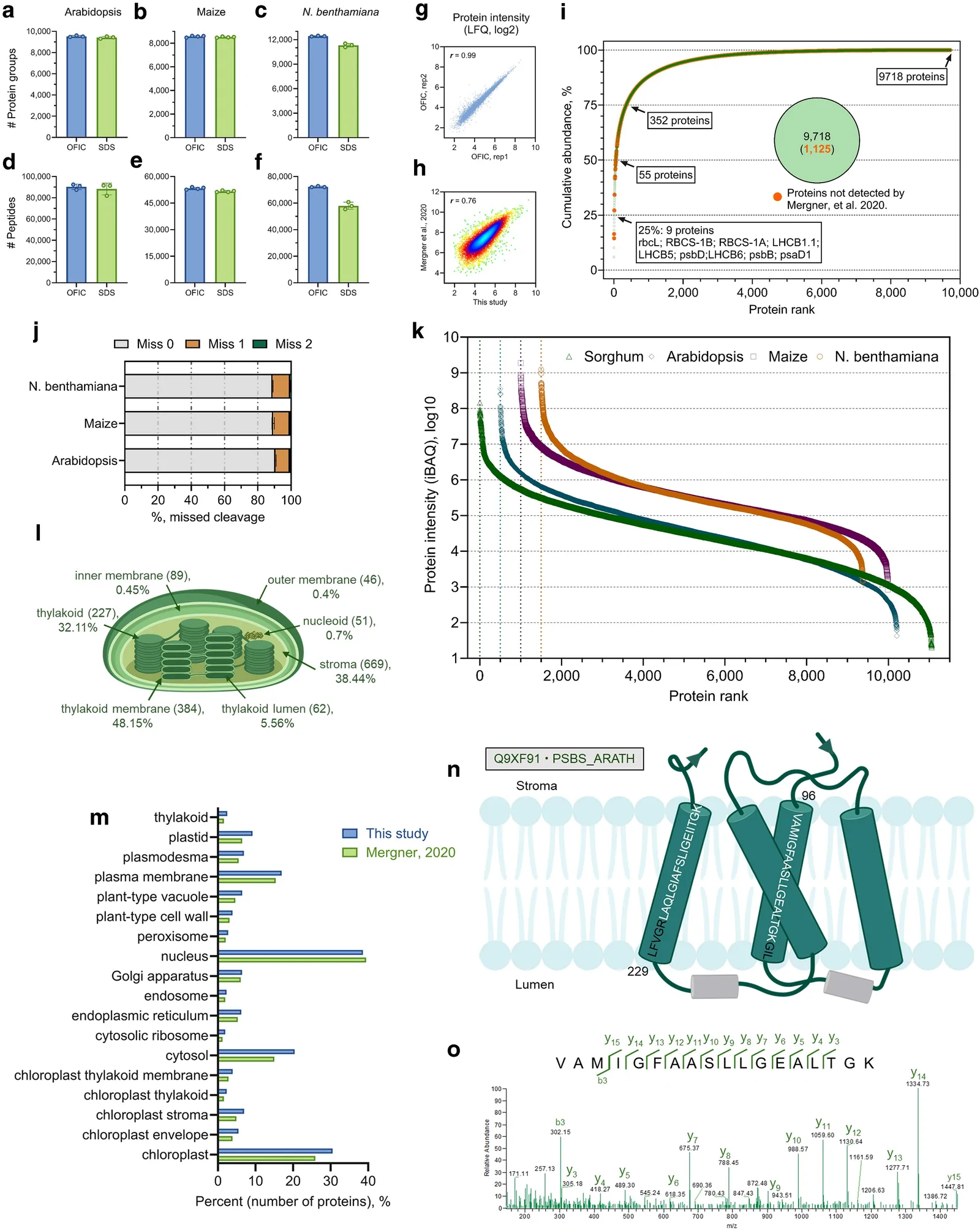
Applying OFIC digestion to proteomics analysis of other plant species. a–c Protein identifications from leaves of *Arabidopsis*, *Maize*, and *N. benthamiana*. d–f Peptide identifications of the 3 types of leaves. g) Reproducibility assessment. Two replicates of OFIC digestion of *Arabidopsis* leaf samples were taken as a representative. Pearson correlation r value is shown in the plot. h) Density plot of the Arabidopsis proteins obtained from this study and the study by Mergner *et al*. Protein group quantity (log2-transformed) was used for the plot. Pearson correlation r value is shown in the plot. i) Cumulative abundance plot of *Arabidopsis* proteome obtained by OFIC digestion. The top 9 proteins in the first quarter (25%) as well as the number of proteins in the other quarters were indicated in the plot. The pie shows the total number of proteins from OFIC method, and the number of proteins that were exclusively identified by OFIC. j) Digestion efficiency assessment of OFIC processing for the 3 types of leaves. k) Dynamic range plot of the proteomes derived from OFIC processing of the 4 types of leaves. l) Quantitative proteomics of *Arabidopsis* chloroplast. Chloroplast proteins and their relative abundance are shown. Numbers in the brackets indicate the number of identified proteins of each category. m) Cellular compartment enrichment analysis of the *Arabidopsis* proteome obtained from this study and the study by Mergner et al. n) Transmembrane protein identification by OFIC method. Protein PSBS is shown as a representative. The peptides that were identified by OFIC are shown as while lines (outside of TM domain) and amino acid sequences (inside TM domain), respectively. A representative MS/MS spectrum is shown in panel o). Some of the panels were created by Biorender.

We next assessed the dynamic range of the *sorghum* leaf proteome derived from OFIC digestion. Taking the median of intensity-based absolute quantification (iBAQ) values of the five replicates, the OFIC approach identified a total of 11,069 *sorghum* proteins, which spanned nearly 7 orders of magnitude (Fig. 3k), and 63 proteins alone accounted for nearly half of the total protein mass (Fig. 2f; Figure S2d). Due to the unsatisfactory performance of TFA lysis, we excluded the TFA-derived proteome from further data analysis. In terms of the overall protein ontologies, both OFIC and SDS methods identified a similar quantity of proteins across a wide range of cellular compartments, such as cytosol, mitochondrion, and nucleolus (Fig. 2e). Proteins related to ribosome and chloroplast showed quantitative variations, although no qualitative differences were seen between the 2 digestion methods (Fig. 2e**, inner panel**). Instead of RuBisCO, the top 5 most abundant proteins in *sorghum* leaf appeared to be ATP synthase subunit alpha (*aptA*) followed by Germin-like protein (*GLP*), Plastocyanin, Cytochrome b559 subunit alpha (*psbE*), and Photosystem II D2 protein (*psbD*). As a C4 plant, these data make sense because *sorghum* is known for its high photosynthetic efficiency and requires fewer RuBisCO enzyme compared to C3 plants such as *A. thaliana* (Sage 2002). Interestingly, these top 5 most abundant proteins were all annotated as chloroplast-localized proteins. The chloroplast is a hallmark organelle in plant cells, playing fundamental roles in photosynthesis, amino acid synthesis, lipid metabolism, and immune responses (Wang et al. 2023b). Our study identified over 500 chloroplastic proteins, which accounted for over 30% of the total protein mass (Fig. 2f**, inner panel**). The data suggest the great dominance of chloroplast proteins in plants, which is not totally surprising. However, the results may still be underrepresented, as only 70% of *sorghum* proteins are annotated in the context of cellular compartment (CC) in the DAVID Knowledgebase. In contrast, over 99% of *A. thaliana* proteins have clear compartmentation annotation in DAVID, and over 3,000 chloroplast-associated proteins were identified in this study (as will be discussed later). Lastly, we examined the subclasses of the chloroplast proteins, in particular the chloroplast membrane proteins. In total, the OFIC digestion data mapped 142 thylakoid membrane proteins as well as 67 chloroplast inner- and outer-membranes, representing the most abundant category of proteins in *sorghum* leaf proteome (22.69%; Fig. 2g). These data corroborate the subcellular localization distribution of the global leaf proteome and underscores the effectiveness of the OFIC method for plant membrane protein identification, despite the rigid protection of the cell wall and potential interference from the massive cellulosic matrix.

### In-cell proteomics analysis of *maize*, *N. benthamiana*, and *A. thaliana* leaves

To investigate whether the in-cell protemoics method has any plant type restrictions or specificities, we further challenged it with three other plants, including *A. thaliana*, *maize*, and *N. benthamiana*. We compared OFIC with the SDS lysate-based method whenever possible. At least 3 biological replicates were prepared for each method. To process leaf samples, we adopted the same procedures as illustrated in Fig. 1. As expected, using a simple in-cell digestion method and a single-shot DIA MS/MS, we were able to identify approximately 9,600 proteins from *A. thaliana*, 8,600 proteins from *maize*, and 12,000 proteins from *N. benthamiana* (Fig. 3a–f; Tables S2, S3, and S4). In line with our previous findings, the OFIC digestion outperformed SDS lysate-based digestion methods again in the context of protein and peptide hits, and the approach is highly reproducible as well (Fig. 3g), ensuring excellent intensity correlation and quantitation accuracy.

Maize leaf proteomics has been studied quite extensively. Over 12,000 proteins have been reported using 2D or 3D LC fractionation and 20 to 40 LCMS injections (Gao et al. 2008; Facette et al. 2013). *N. benthamiana* leaf proteomics applications appeared to be relatively limited regarding the depth, covering only 6,000 proteins or less with LC or gel-based pre-fractionation (Shen et al. 2022; Zheng et al. 2024a). *A. thaliana* is the foremost model plant species with a well-annotated genome. Mergner and coworkers reported the identification of approximately 15,000 *A. thaliana* leaf proteins using exhaustive sampling strategies (ie, multi-shot LCMS with 24 fractions) (Mergner et al. 2020). With single-shot LCMS, Mikulášek *et al*. reported around 5,000 protein hits derived from SDS lysate of whole *A. thaliana* leaf (Mikulášek et al. 2021). In this study, we utilized a much simpler and more rapid approach but achieved equivalent or deeper proteome coverage. Regarding the effectiveness of in-cell digestion, we observed generally good to excellent efficiency for all 3 types of leaves (Fig. 3j), which is consistent with the digestion data from *sorghum* leaf described above and *C. elegans* worms reported recently (Elsayyid et al. 2025). Meanwhile, massive overlaps can be seen between the proteins obtained from in-cell digestion and the proteins derived from lysate-based processing, either in this study or from other studies (Figure S3a–d). Quantitatively, both the *maize* and the *N. benthamiana* proteome span over six orders of magnitude (Fig. 3k), suggesting broadly deep coverage of these 2 hard-to-lyse leaf tissues by the in-cell proteomics approach. On the other hand, comparing the *A. thaliana* data obtained from this study and Mergner's (Mergner et al. 2020), we also observed generally good correlation and comparable distribution of subcellular localizations, with minor variations from cytosolic, chloroplastic, and mitochondrial proteins (Fig. 3h and 3m). In summary, these data suggest minimal quantitative bias of the in-cell proteomics approach and demonstrate the broad applicability of the in-cell digestion method for proteomic analysis of plant leaves.

We then took a further look at the OFIC-derived *A. thaliana* leaf proteome, which spans nearly 7 orders of magnitude, and agrees well with previous reports (Mergner et al. 2020). According to DAVID annotation, approximately 3,050 proteins were classified as chloroplastic proteins, which was the second largest category (slightly less than the 3,800 nuclear proteins) and accounted for over 78% of total *A. thaliana* leaf protein mass. Interestingly, 384 chloroplast thylakoid membrane proteins, as classified by DAVID database, contributed to nearly half (48.2%) of the overall leaf protein mass (Fig. 3l). Among the membrane proteins, the transmembrane (TM) protein subfamily is notoriously tough for proteomic identification. Of the 384 classified chloroplast thylakoid membrane proteins, 121 were predicted by TMHMM 2.0 to have at least 1 transmembrane helix, highlighting the potential of the in-cell strategy for analyzing membrane proteins as well as TM proteins. As a representative, photosystem II 22 kDa protein (PSBS) is a multipass chloroplast thylakoid membrane protein. With in-cell digestion, 7 peptides were identified for this protein, including 2 sequences in the stroma region, 3 in the lumen region, and another 2 in the transmembrane regions (TM1 and TM4; Fig. 3n and o). There was clear evidence that the cleavages occurred at the embedded sites in the lipid bilayer. These data support our hypothesis that methanol fixation may solubilize phospholipids and facilitate the tryptic cleavage, thereby enabling successful identification of integral membrane proteins. In the context of membrane protein identification, we further examined the OFIC- and SDS-derived proteomes globally, through a comparative analysis of the protein- and peptide-level hydrophobicity properties (Figure S4). Our data suggested that the in-cell digestion method is unbiased for hydrophobic protein analysis compared to detergent-based extraction. From a quantitative standpoint, the 3 most abundant proteins in *A. thaliana* leaves are RuBisCO enzymes (RbcL, RbcS-1B, and RbcS-1A) that contributed to over 14% of the total protein mass (Fig. 3i), which is expected for a C3 plant such as *A. thaliana*. The overwhelmingly high abundance of RuBisCO proteins has been a major challenge for plant leaf proteomics, and depletion or fractionation strategies has been developed to facilitate identifying the low-abundance proteins (Gupta et al. 2015; Tola and Missihoun 2023). Here, our data showed that even without RuBisCO depletion, the “in-cell proteomics” strategy in combination with DIA LCMS is capable of deep proteome analysis of plant leaves.

Lastly, we asked if and to what extent the manual slicing and methanol fixation, the 2 major pretreatment steps of the “in-cell proteomics” strategy, could induce wound- or stress-related artifacts and thereby alter the plant leaf proteome. To address this, we performed independent experiments comparing proteome-wide changes caused potentially by the OFIC and cryogenic grinding-based workflows (Figure S5). Grinding fresh leaf tissue with liquid nitrogen (LN) is a common practice by the plant proteomics community as it rapidly halts biological activity, preserves the physiological state of the tissue, and enables efficient lysis and protein extraction for downstream analysis. Based on the comprehensive head-to-head comparisons (Cut_OFIC vs. LN_OFIC) (Figure S6 and Table S5), we did not observe significant qualitative and quantitative differences between them in terms of the number of protein and peptide hits, identification uniqueness, technological reproducibility, differential protein expression and protein subcellular localizations (Figure S6). We further compared it with 2 additional protocols that were examined in parallel, one involved prefixing fresh leaves with methanol prior to manual slicing (MetOH_OFIC), and the other combined LN flash-freezing and grinding with sequential methanol fixation and SDS lysis (LN_SDS) (Figure S5). Because methanol-fixed cells undergo immediate lysis upon exposure to SDS, the latter protocol specifically tested whether coupled fixation and detergent lysis enhances overall yield independently of enzymatic accessibility. Reassuringly, comparison across these diverse conditions revealed no systematic bias or artifacts introduced by the OFIC workflow (Figure S6). These findings are consistent with a recent report (Chen et al. 2026), which demonstrated that alcohol-based fixation preserves both basal and drug-induced proteome states in HeLa cells when compared with snap-freezing, supporting the general robustness of alcohol fixation for proteome preservation. Collectively, our results indicate that neither manual slicing nor methanol fixation introduces detectable global proteomic bias under the conditions tested. Therefore, when liquid nitrogen is not readily available, rapid tissue slicing followed by direct in-cell processing represents a practical and reliable alternative for plant proteomic analysis.

### Applying in-cell proteomics to plant pollen and seeds

**Figure 4:**
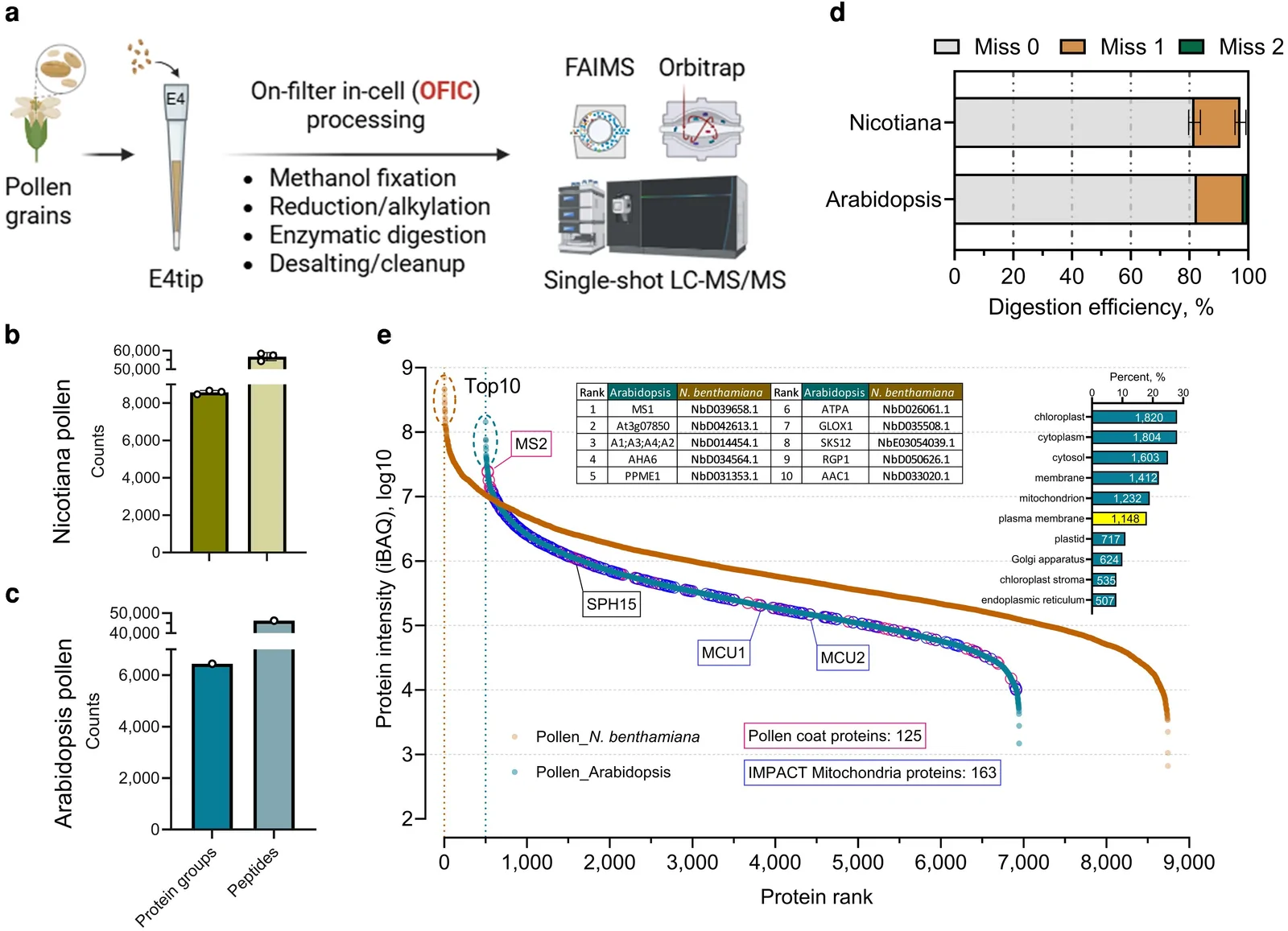
In-cell proteomics analysis of plant pollen. a) Illustrative workflow. b–c Protein and peptide identifications from *Nicotiana* pollen and *Arabidopsis* pollen, respectively. d) Digestion efficiency assessment. e) Protein ranking plot. Inner table shows the top 10 most abundant proteins in the 2 plant pollen proteomes. The inner bar diagram shows the top 10 most enriched cellular compartment categories. Panel A was created by Biorender.

With the systematic justification of the in-cell approach for leaf proteomics, as shown above, we next asked if the approach can be used to process nonleaf plant tissues. We tested pollen grains and seeds in this regard to demonstrate the versatility and wide applicability of the approach. Regarding the filter device, we chose tip-based filters to minimize potential sample loss for plant tissues with typically limited availability. Previously, pollen proteomics studies required the grains to be ground or homogenized, in order to break the rigid pollen coat and extract proteins for subsequent digestion experiments (Dai et al. 2007; Darnhofer et al. 2021). The procedures are time-consuming, and often require large inputs. Here, we speculate that pollen grains are micron-scale particles; hence, they may be processed using E4tips, similar to mammalian cells and *C. elegans* worms we examined recently (Elsayyid et al. 2025; Moafian et al. 2025). Therefore, based on sample availability, we used approximately one-tenth of the pollen grains collected from a single *N. benthamiana* flower and grains pooled from 2 *A. thaliana* flowers for one digestion experiment, and we performed triplicate digestion experiments with *N. benthamiana* pollen, and one replicate with *A. thaliana* pollen. With the “all-in-one” tip, surprisingly again, we were able to identify approximately 8,500 and 6,400 proteins from *N. benthamiana* and *A. thaliana* pollen, respectively (Fig. 4b and c; Tables S6 and S7). The digestion efficiency was relatively lower in the pollen samples compared to the leaf samples, but still greater than 80% of the peptides were completely digested (Fig. 4d), outperforming some of the commonly used filter-based digestion approaches (Ludwig et al. 2018).

To fully justify the in-cell approach as a valid alternative to pollen proteomics, we performed independent head-to-head comparisons with a lysate-based digestion method (Figure S7a). Using roughly a quarter aliquot of the pollen grains from one *N. benthamiana* flower, both methods could detect approximately 9,400 proteins in duplicate digestion experiments, and over 99% of them were shared (Figure S7b and S7d; Table S8). On peptide level, the OFIC approach performed slightly better, as evidenced by slightly more peptides, higher total intensity, and fewer miscleavages (Figure S7c, e and j). Quantitatively, although both methods showed excellent correlation between replicate digestion experiments (Pearson ***r*** > 0.95; (Figure S7k–m), the OFIC approach provided lower variabilities on either protein or peptide level (Figure S7g, h). Overall, the 2 processing methods provided very similar quantitative pollen proteomes, as only a few significantly differential proteins were seen between them (Figure S7, f and i). These data demonstrate the unbiased nature of the in-cell approach for pollen proteomics analysis.

Pollen methionine synthase 1 (MS1) is a plant enzyme that synthesizes methionine for pollen development, particularly for the activation of calcium channels important for seed germination and pollen tube growth. Interestingly, MS1 protein was the most abundant protein in both *A. thaliana* and *N. benthamiana* pollens we investigated (Fig. 4e). Noteworthy is that many (approximately 42%) of the pollen coat proteins reported in a recent study (Wang et al. 2023a) were also identified by the OFIC approach such as MS2, an essential component of the pollen wall involved in the synthesis of fatty alcohols. Pollen development is extremely energy-demanding, and mitochondrial proteins are thought to play crucial roles in this process. A recent proteomics study of enriched mitochondria using IMPACT strategy identified approximately 200 proteins enriched in pollen mitochondria (Boussardon et al. 2025), and the OFIC approach detected 80% of them, highlighted by mitochondrial calcium uptake 1 and 2 proteins (MCU1, MCU2). A broader GO enrichment analysis suggested that nearly 1,148 pollen proteins (approximately 18%) were annotated as plasma membrane proteins. These data underscore again the power of the in-cell proteomics approach, which can cleave proteins effectively in intact pollen grains.

**Figure 5:**
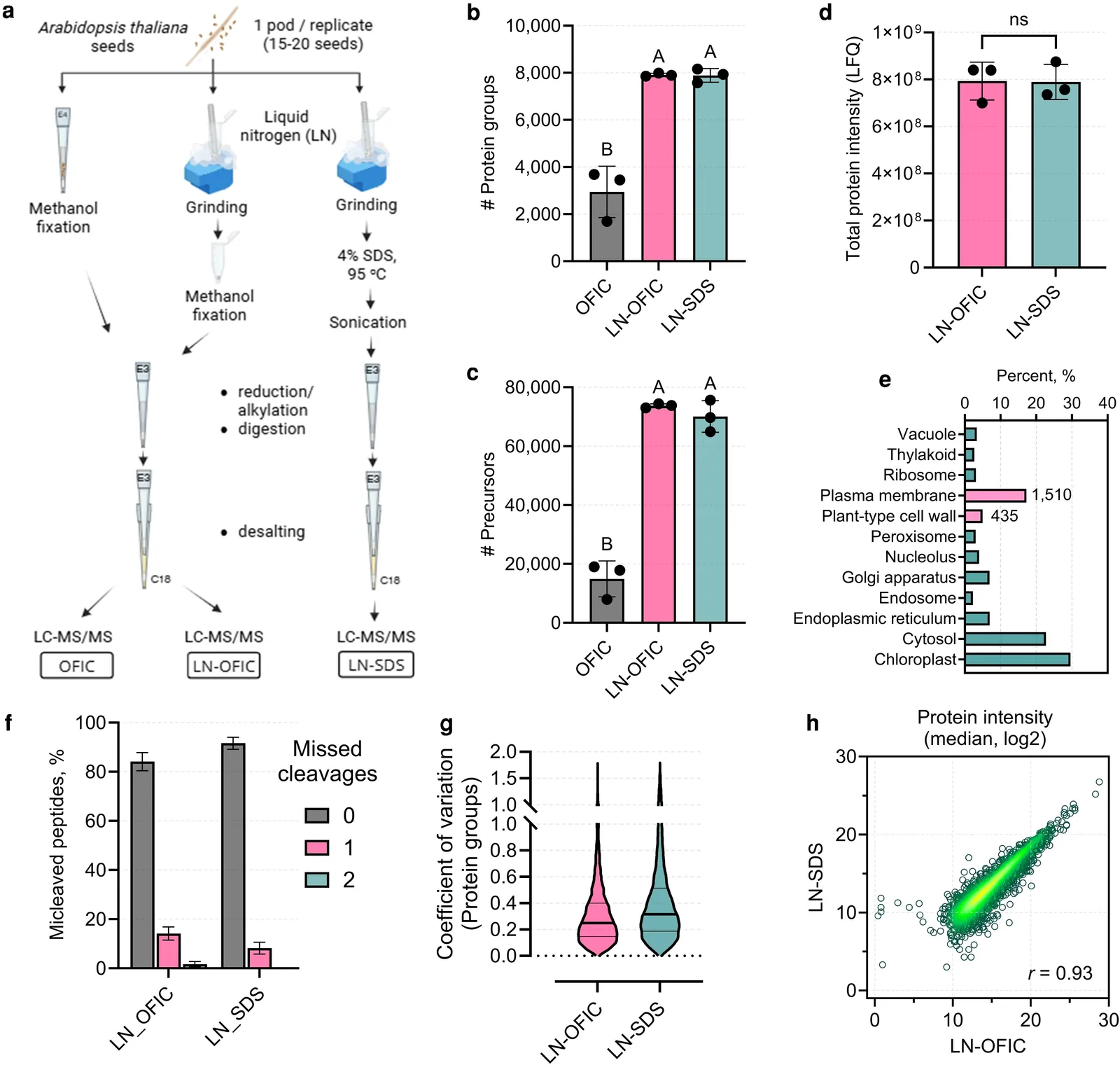
In-cell proteomics analysis of plant seeds. a) Illustrative workflow of different processing methods for *A. thaliana* seeds. b–c) Protein and peptide identifications. Three replicate digestion experiments were performed for each method. Ordinary one-way ANOVA *t*-test and compact letter display were applied. d) Summed protein intensity. e) Subcellular analysis of proteins derived from LN-OFIC method. f) Digestion efficiency assessment. g) Coefficient variation of protein groups. h) Pearson correlation analysis with median protein intensity values of each method. Panel A was created by Biorender.

Plant seeds stand out as an extreme challenge to the “in-cell proteomics” approach. Basically, when processing intact whole seeds, around 15 to 20 derived from one *A. thaliana* pod (or silique) with the OFIC method (Fig. 5a), severely compromised peptide signals and large variabilities were seen, ranging from 1,600 to 3,700 proteins and 7,000 to 16,000 peptides in triplicate digestion experiments. By contrast, when processing ground seeds, OFIC achieved remarkably more hits, averaging nearly 7,900 proteins and 60,000 peptides (Table S9). The identification rates were not only reproducible between the replicates but also consistent with a SDS lysate-based digestion experiment carried out in parallel (Fig. 5b–c). The data highlight that the seed coat might represent a much stronger physical barrier than the leaf cell wall or pollen wall, hindering sufficient penetration or permeabilization by the fixation agent such as methanol. As it turned out that the in-cell approach does not work optimally for seeds, we suspect that it may suffer as well in some other hard tissues not studied here, such as plant stem and root tissues. In this regard, our data suggested an immediate alternative, that is, simply grinding the tough tissues in liquid nitrogen followed by in-cell treatment can offer equivalent performance to lysate-based seed proteomics. More specifically, after disrupting the seed walls, methanol can treat the exposed cellular components as effectively as other plant tissues we investigated above, leading to similar summed protein intensities, better reproducibility, fewer variations, and overall equivalent quantitative proteome profile to the SDS lysate-based digestion, as only a few significantly differential proteins were seen between the 2 proteomes (Fig. 5 and Figure S8). Regarding the digestion efficiency, although the OFIC approach cleaved on average 84% of the total peptides completely (Fig. 5f), which was lower than the SDS lysate method tested here (approximately 91%), it was still on similar levels (80 to 90%) with the digestion efficiency of other plant tissues by the OFIC approach as described above.

In terms of the *A. thaliana* seed proteome, it spanned nearly 7 orders of magnitude, and the top 10 most abundant proteins accounted for almost 48% of the total amount of proteins (Figure S8a and c). Among them, 4 were seed storage proteins (CRC, CRA1, AT2S2, and CRB) and 4 were oleosins (OL2, OLEO1, OLEO2, and OLEO5), the latter of which are important for controlling oil body structure and lipid accumulation in *Arabidopsis* seeds (Siloto et al. 2006). These data were in line with previous findings (Mergner et al. 2020). Despite the overwhelmingly high abundance of these tissue proteins, the in-cell approach can still probe the seed proteome deeply. Regarding the protein classifications, over 1,800 of them were annotated as cell wall or plasma membrane proteins, which contributed to almost 19% of the total protein intensity (Fig. 5e and Figure S8c). Taken together, the extensive comparison data with lysate-based processing method proved that the “in-cell proteomics” approach is simple yet equivalently powerful, and could be employed to investigate plant leaf, pollen, and seed biology broadly.

### Proteomic investigation of *N. benthamiana* leaves inoculated with *Colletotrichum*

**Figure 6:**
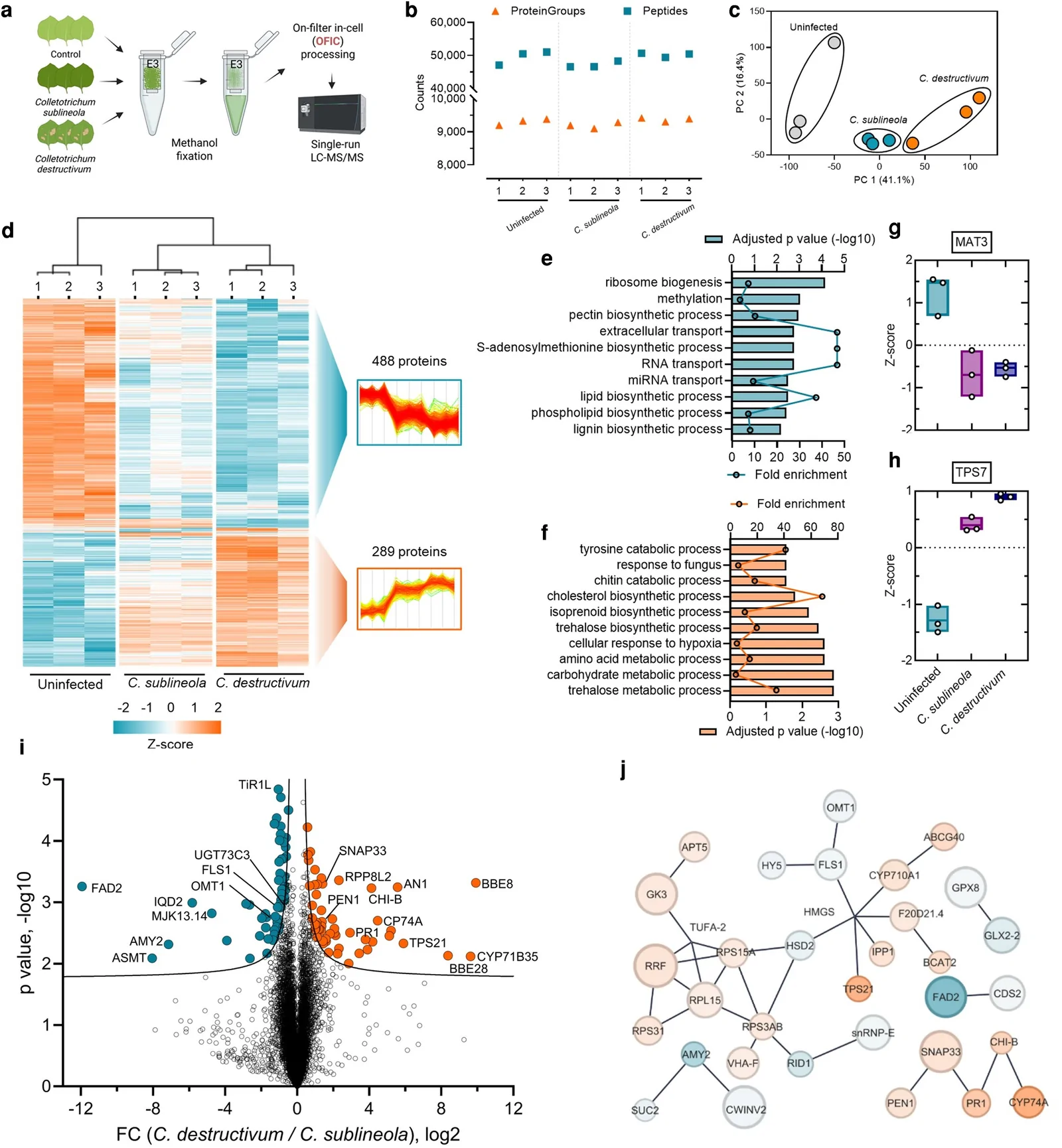
Applying in-cell proteomics to examine plant infection and immune response. a) Experimental flowchart. Created with BioRender.com. b) Protein and peptide identifications of the 3 groups. Three biological replicates were included for each group. c) Principal component analysis. d) Heatmap of ANOVA significant proteins (Permutation FDR 0.01, S0 = 0.5) among the 3 conditions. The proteins have been filtered out of protein identified by single peptides. e–f) Gene Ontology enrichment analysis of the 2 clusters highlighted in the heatmap. g–h) Two representative proteins that showed opposite patterns upon infection. i) Volcano plot. The pairwise *t*-test (FDR 0.05, S0 = 0.1) of the 2 infected groups. j) Protein network analysis. The differentially expressed proteins that showed significance in panel I were used to build the STRING network in Cytoscape. Panel A was created by Biorender.

Having established a robust “in-cell proteomics” workflow for rapid and deep proteome analysis of plant tissues, we next sought to provide experimental evidence of this workflow for answering critical biological questions. For this purpose, we inoculated the *N. benthamiana* leaves with 2 strains of fungus that exhibit different host specificity, one was the nonhost-adapted *C. sublineola* that can invoke nonhost resistance (NHR), and the other was the host-adapted *C. destructivum* that infects and causes anthracnose disease. We processed them for proteomics analysis using the in-cell approach described above (Fig. 6a; Table S10). Experimentally, we identified on average 9,300 proteins from each of the 3 replicates per group, totaling approximately 9,500 proteins with minimal missing values and high reproducibility (Fig. 6b; Figure S9a, b). Interestingly, although the leaf proteomes displayed largely similar patterns upon infection, their global profiles were already distinguishable according to a straightforward principal component analysis (Fig. 6c), suggesting that the single-run, in-cell proteomics approach was able to capture subtle proteome-wide alterations upon fungal inoculation.

We looked further into the quantitation data to learn more about the molecular defense mechanisms and metabolic alterations in response to fungus infection. A multisample ANOVA test (*P* < 0.05) led to approximately 2,800 significantly differentially expressed proteins among the 3 groups, indicating a system-level proteome remodeling triggered by the infection (Fig. 6c). We then narrowed down the candidates utilizing a more stringent cutoff (Permutation FDR 0.01, stabilizing constant S0 = 0.5) for comparison, and also required the candidates to be matched by at least 2 unique peptides. The analysis resulted in 819 significant proteins among the 3 conditions, which were grouped into 2 major clusters, with 488 proteins in 1 cluster showing a broad decline in expression upon fungal inoculation, and 289 proteins in the other showing up-regulation (Fig. 6d). Multiple metabolic processes were found to be up-regulated upon fungus infection. Among them, the trehalose metabolic process showed the highest significance (with high fold-enrichment). Trehalose is a sugar that supports fungal pathogens infection of plants by promoting appressorium development and maintaining cellular turgor, which are crucial for penetrating plant tissue (Foster et al. 2003). Ten trehalose-phosphate synthase proteins were identified from this experiment, and 4 of them showed a significant increase, including TSP10, TSP11, TSP6, and TSP7 (Fig. 6f). The down-regulated proteins are mainly involved in ribosome biogenesis, methylation, and pectin biosynthetic process. While methylation can influence ribosome biogenesis by altering the expression of key nucleolus proteins, its declined expression may imply a reduced level of protein translation or synthesis. This finding was corroborated by the decline of the S-adenosylmethionine (SAM) biosynthetic process. The adenosyltransferase (MAT) proteins play crucial roles in one-carbon metabolism, providing the methyl group for transmethylation reactions (Hanson and Roje 2001). At least 3 MAT proteins were mapped in our study, including MAT3 (or SAMS3; Fig. 6e), which has been reported previously to be targeted and manipulated by certain plant pathogens to weaken the host's defense system and promote infection (Liu et al. 2014; Zheng et al. 2024).

Next, we examined how *N. benthamiana* plants responded differently when challenged with host-adapted *C. destructivum* or nonhost-adapted *C. sublineola* (Fig. 6i). Interestingly, the two-sample *t*-test revealed 2 O-methyltransferase (OMT) proteins, which are involved in one-carbon metabolism, (Lam et al. 2019) were downregulated in plants infected with *C. destructivum.* An acetylserotonin OMT (ASMT) was one of the most down-regulated proteins and homologs are involved in the production of melatonin, which is a potent antioxidant implicated in stress responses (Back 2021). A 3-O-methyltransferase (3-OMT) was also down-regulated and orthologs in tomato (sh3-OMT) and *A. thaliana* (atOMT1) can catalyze 3′-O-methylation of flavonols, such as myricetin (Schmidt et al. 2012; Lam et al. 2024). A STRING pathway analysis of differentially expressed proteins in *C. destructivum and C. sublineola* inoculated plants found that OMT1 belongs to a diverse group of biosynthetic proteins (Fig. 6j), but is directly linked to flavonol synthase 1 (FLS1), a key enzyme in the flavonoid biosynthetic pathway (Owens et al. 2008). Thus, although both fungi down-regulated MATs, these data suggest that the nonhost-adapted *C. sublineola*, and thus noninfectious, cannot downregulate these OMTs, potentially pointing to an NHR mode of action to prevent infection.

Multiple proteins related to jasmonic acid (JA) defense pathway were upregulated in leaves infected with *C. destructivum.* These include an allene oxide synthase (CYP74A), which plays a crucial role in the plant's defense against infection by initiating the biosynthesis of JA (Hughes et al. 2014; Svoboda et al. 2021). JA is a key signaling hormone that activates defense responses that includes the production of antifungal compounds, such as terpenoids and chitinases. The terpenoid biosynthetic enzyme, 5-*epi*-aristolochene synthases-like protein (EAS-like), also increased in response to *C. destructivum* and related EASs produce the antifungal terpenoid, phytoalexin capsidase (O’Maille et al. 2006). The basic chitinase (CHI-B), which is known as pathogenesis-related protein 3 (PR-3), was also upregulated in *C. destructivum* infected plants and is a well-studied antifungal enzyme that directly hydrolyzes chitin in fungal cell walls (Rawat et al. 2017). All 3 proteins were confidently identified by more than 7 peptides, and showed significant increase upon *C. destructivum* infection (Fig. 6g), which is consistent with previous findings. The STRING analysis (Fig. 6j) showed that CYP74A and CHI-B belong to a defense-related group that also has pathogenesis-related protein 1 (PR1) (Mei et al. 2006). Interestingly, that group also has a SNAP25 homologous protein (SNAP33) and SYP122 syntaxin (also known as PEN1), which is a well-known fungal defense related protein (Assaad et al. 2004) found in extracellular vesicle (Rutter and Innes 2017). SNAP33 and PEN1 are both implicated in vesicle-mediated secretion, and are vital components of a well-studied exocytic pathway for host-specific fungal disease resistance SNARE complex (Wang et al. 2016). This cluster suggests that a coordinated hormone signaling coupled exocytosis is required to reinforce pathogen restriction at the cellular interface in the plant host defense response (Yun et al. 2016).

## Conclusions

In this benchmark study, we systematically evaluated the feasibility of an on-filter in-cell proteomics strategy for plant tissue sample preparation. Through systematic comparisons with conventional cryogenic grinding and lysate-based workflows, we demonstrated that the OFIC digestion approach provides an easy-to-use and streamlined alternative while maintaining the ability to process traditionally challenging plant tissues with high proteome depth and sensitivity. We first established the applicability of this strategy to leaf proteomics across multiple models and crop plant species, including *A. thaliana, N. benthamiana, sorghum,* and *maize*, and subsequently extended the workflow to additional plant tissue types, including pollen and seeds. Using single-run DIA analyses with minimal input materials, the OFIC workflow enabled the identification of approximately 9,000 to 12,000 proteins from leaf tissues, 7,000 to 9,000 proteins from pollen, and approximately 8,000 proteins from seeds. Despite completely eliminating protein extraction steps, the OFIC approach achieved digestion efficiency, proteome coverage, detection sensitivity, and quantitative accuracy comparable to those of conventional lysate-based methods across all sample types examined. Notably, the workflow also obviated the need for depletion of highly abundant proteins such as RuBisCO and photosystem-associated proteins, thereby further simplifying plant proteomics sample preparation. These results demonstrate the potential of OFIC to substantially reduce the technical complexity associated with conventional plant proteomics workflows (eg, protein precipitation, re-solubilization, etc.). Given its versatility, this approach may be readily extended to other plant tissues, including siliques, flowers, embryos, and cultured plant cells. At the same time, our results highlighted certain practical limitations of the OFIC approach. For instance, analysis of *A. thaliana* seeds revealed that intact seeds could not be processed directly by the in-cell workflow; disruption of the seed coat was required to permit enzymatic access, although protein lysis and extraction remained unnecessary. This observation suggests that physical barriers, rather than protein accessibility itself, may represent the primary constraint for OFIC-based sample preparation in some plant tissues. Consequently, for exceptionally rigid tissues such as roots and stems, partial mechanical disruption—for example, cryogenic grinding in liquid nitrogen—followed by OFIC processing may provide a practical compromise that preserves the simplicity of the workflow while enabling comprehensive proteome analysis. Collectively, our findings establish OFIC as a robust, scalable, and broadly applicable strategy for plant proteomics and provide a foundation for its future application across diverse plant species and tissue types.

The “in-cell proteomics” technology was diversified by using different types of filter devices. In this study, for chlorophyll-rich tissues such as green leaves, we employed E3 filters, which exhibit minimal binding to chlorophyll and effectively deplete pigments. This minimizes potential interference with downstream LCMS analysis, even without traditional trichloroacetic acid (TCA)/acetone precipitation (Isaacson et al. 2006). E3 filters also feature relatively large loading capacities, enabling the processing of up to approximately 20 cm^2^ of leaf tissue, sufficient for typical shotgun proteomics analyses and some post-translational modification enrichment. For preparative applications requiring even larger amounts of starting material (ie, pooling of multiple leaves), E3 cartridges (1 mL or 3 mL volume size) provide a convenient alternative. For high-throughput workflows involving large sample sets (eg, >100 samples), the multiwell format E3 plate is an efficient and ready-to-use solution. Previous studies have demonstrated the successful application of these devices for bacterial cells and body fluids, respectively (Martin et al. 2024). For pollen grains, which typically contain few pigments, the E4 filter is a viable option. It is an enhanced version of the E3 technology, incorporating C18-based functionality to improve peptide retention and cleanup. The E4 tip, in particular, is highly advantageous for small sample inputs due to its low reaction volume and integration of all sample preparation steps into a single device. This design makes it well suited for low-cell or even single-cell plant proteomics applications (Montes et al. 2024).

From a technological perspective, it is worth noting that the OFIC digests generated in this study were analyzed using FAIMS and DIA MS, both of which are known to increase the proteome coverage relative to classical shotgun proteomics lacking FAIMS, or employing data-dependent acquisition (DDA) MS. For instance, FAIMS can reduce chemical background, particularly from singly charged ions, while providing an orthogonal gas-phase separation dimension prior to mass spectrometric analysis. Several studies have reported substantial improvements in peptide and protein identification rates using FAIMS, often in the range of 30% to 50% depending on sample type and experimental design (Hebert et al. 2018; Pfammatter et al. 2018). Likewise, DIA generally provides improved quantitative reproducibility and sensitivity compared with DDA and is particularly advantageous for the detection and quantification of low-abundance proteins. For instance, Khalil *et al*. reported that DIA enabled the identification of nearly 45% more proteins than DDA for host cell protein analyses (Khalil and Plisnier 2025). In this regard, we speculate that the deep proteome coverage achieved in this study likely reflects the combined benefits of streamlined single-vessel OFIC sample preparation, FAIMS-based gas phase fractionation, and DIA MS analysis. While the present study was not designed to dissect the individual contributions of these components, their synergistic effects likely facilitated the comprehensive profiling of plant leaf, pollen, and seed proteomes. Importantly, because the OFIC workflow can generate LCMS-compatible peptide samples in an extremely simple and rapid fashion (ie, totaling 20 min or less centrifugation time compared to around 240 min by FASP) (Song et al. 2018), this workflow should be readily adoptable by future advances in MS instrumentation. We therefore anticipate that even deeper proteome coverage could be achieved by coupling OFIC with next-generation platforms such as timsTOF- or Astral-based mass spectrometers. Indeed, in a few benchmark studies of these newest MS instruments, data have shown that when injecting the same protein digests to Astral Orbitrap or other previous generations, Astral can report over 2 times more proteins and 3.5 times more peptide precursors than Orbitrap Exploris 480 MS instrument, even when using shorter LC gradients (Guzman et al. 2024). When compared with Tribrid instruments such as Ascent or Eclipse Orbitrap in the context of TMT-based quantitation, Astral can quantify 50% more peptides and 20% more proteins (He et al. 2025). These advances suggest that the proteome depths reported here may represent only a fraction of what could ultimately be achieved through the integration of OFIC with emerging MS technologies.

Finally, we applied the in-cell approach to investigate complex biological processes such as the differences in plant defense responses to nonpathogenic *C. sublineola* and pathogenic *C. destructivum*. Although proteomics has been widely employed to study plant-microbe interactions, there are very few studies comparing nonhost responses to pathogen infection. NHR is likely a combination of physical barrier defenses, pathogen-associated molecular pattern-triggered immunity, antimicrobial compounds, and effector-triggered immunity. Proteomic analysis of NHR has been limited to a few studies that compare NHR of a nonpathogenic microbe to a mock inoculation control (Li et al. 2012; Dong et al. 2015). In this study, we performed a 3-way comparison among the NHR of *C. sublineola*, *C. destructivum* infection, and the mock inoculation control. Surprisingly, PEN1, which was originally identified as a key NHR component (Nürnberger and Lipka 2005) was more upregulated in *C. destructivum* infected plants than *C. sublineola* infected plants undergoing NHR. However, PEN1 has important roles beyond NHR in effector triggered immunity (Johansson et al. 2014) and extracellular vesicles (Rutter and Innes 2017). Our study also discovered intriguing differences in one-carbon metabolism and implicates OMTs involved in melatonin and flavanol production, which opens a potential future avenue of research on how they may contribute to NHR.

In conclusion, our results demonstrate the feasibility, versatility, and biological utility of “in-cell proteomics” for plant systems and establish OFIC as a streamlined alternative to conventional plant proteomics workflows. We anticipate that the developed approach in this study will allow a broad range of researchers, in addition to plant biologists, to probe complex biological questions in even some of the most challenging tissue types, such as pollen and seeds.

## Supplementary Material

kiag557_Supplementary_Data

## Data Availability

The MS raw data and Spectronaut result files (.sne format) associated with this study have been deposited to MassIVE server (https://massive.ucsd.edu/) via identifier MSV000097786.
