# Supplementary material for: Toward simple, rapid, and deep plant proteome analysis with an in-cell proteomics strategy
Source: Plant Physiol. 2026 Jul 30;202(1):kiag557. doi: 10.1093/plphys/kiag557 (PMC13544443; doi:10.1093/plphys/kiag557)
Supplement: Supplementary file 1 [file kiag557_supplementary_data.zip › Supplementary Information_20260531.pdf]

Supplementary Information

Toward simple, rapid, and deep plant proteome analysis with an in-cell proteomics strategy

Deji Adekanye[1], Jasmine Parks[2], Meghana Kusuru[3], Jeffrey L Caplan[1,4,*#], Yanbao Yu[2,*#]

1. Department of Biological Sciences, University of Delaware, Newark, DE, USA
2. Department of Chemistry and Biochemistry, University of Delaware, Newark, DE, USA
3. Department of Computer and Information Sciences, University of Delaware, Newark, DE, USA
4. Department of Plant and Soil Sciences, University of Delaware, Newark, DE, USA

* Authors for communication: jcaplan@udel.edu; yybyu@udel.edu.

# Equal contribution

Table of Content

Supplementary Figure S1. Microscopic assessment of sorghum leaf upon fixation.

Supplementary Figure S2. Evaluation of OFIC digestion for sorghum leaf proteomics.

Supplementary Figure S3. Venn diagram analysis.

Supplementary Figure S4. Overlapping, hydrophobicity and transmembrane analyses of OFC and SDS-base processing methods for leaf proteomics analysis.

Supplementary Figure S5. Illustrative workflow of the proteomic comparison of OFIC with grinding-based strategies.

Supplementary Figure S6. Quantitative assessment of different processing methods for *A. thaliana* leaf proteomics.

Supplementary Figure S7. Quantitative assessment of different processing methods for *N. benthamiana* pollen proteomics.

Supplementary Figure S8. Quantitative analysis of *A. thaliana* seed proteome.

Supplementary Figure S9. Quantitative analyses of plant leaf infection.

Supplementary Protocol 1. In-cell proteomics analysis of plant leaf using E3filter

Supplementary Protocol 2. In-cell proteomics analysis of plant pollen using E4tip

Supplementary Protocol 3. In-cell proteomics analysis of plant seeds using E3tip

Supplemented separately

Supplementary Table S1. Sorghum leaf proteome.xlsx

Supplementary Table S2. Arabidopsis leaf proteome.xlsx

Supplementary Table S3. Nicotiana leaf proteome.xlsx

Supplementary Table S4. Maize leaf proteome.xlsx

Supplementary Table S5. Arabidopsis leaf proteome_comparison with grinding.xlsx

Supplementary Table S6. Arabidopsis pollen proteome.xlsx

Supplementary Table S7. Nicotiana pollen proteome.xlsx

Supplementary Table S8. Nicotiana pollen proteome_comparison with SDS.xlsx

Supplementary Table S9. Arabidopsis seed proteome.xlsx

Supplementary Table S10. Nicotiana leaf infection proteome.xlsx

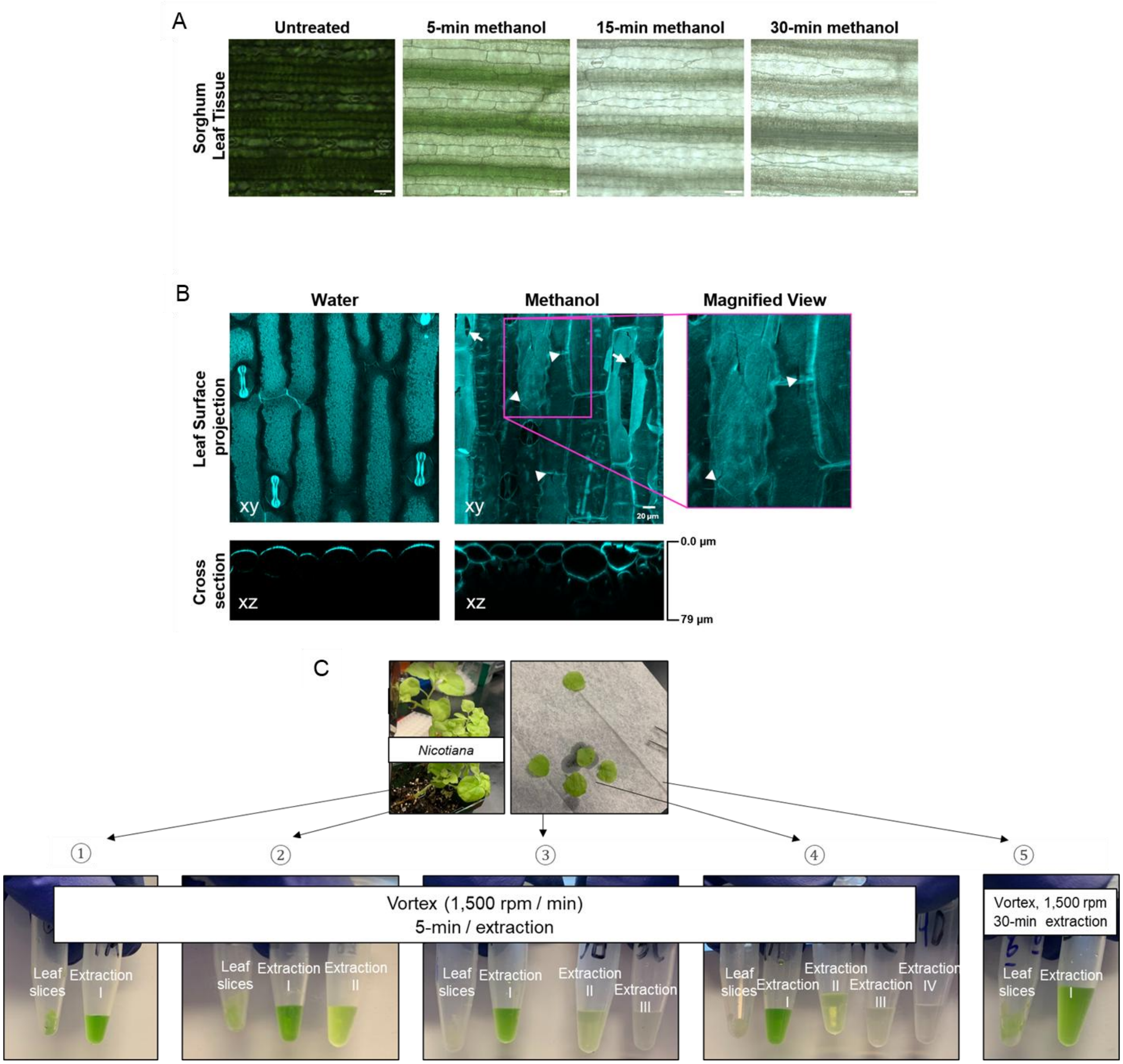

Supplementary Figure S1. Microscopic assessment of sorghum leaf upon fixation. (A) Brightfield images showing the effect of 0, 5, 15, and 30 minutes of methanol treatment on leaves of *Sorghum bicolor*. Scale bar equals 50 µm. (B) Sorghum leaf sections treated with water or methanol, labeled with the cell wall dye Calcofluor White MR, and then imaged using multiphoton microscopy. Maximum intensity projections of the leaf surface (XY) show small cracks along the cell wall junctions (arrowheads) and larger breaks in the cell walls (arrow). A cross-sectional view (XY) comparing the staining and imaging depth between water and methanol cleared samples. Scale bar equals 20 µm. (C) *Nicotiana benthamiana* leaves after clearing with 1, 2, 3, or 4 exchanges (①-④) of methanol compared to a single 30-minute methanol treatment (⑤).

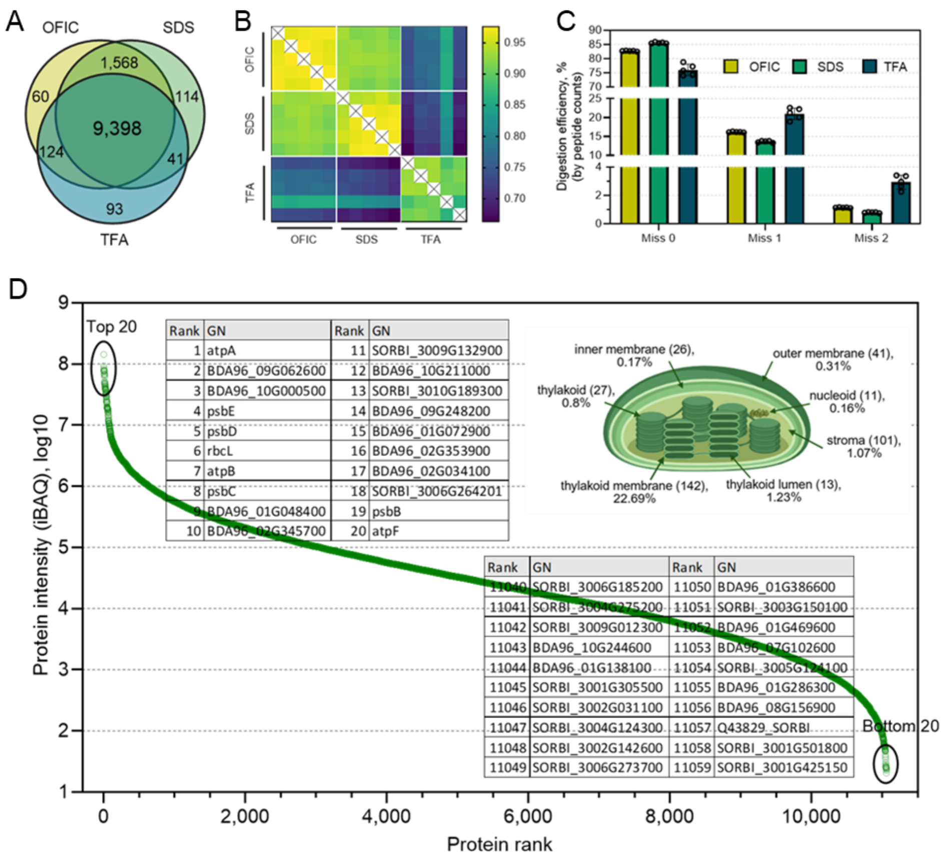

Supplementary Figure S2. Evaluation of OFIC digestion for sorghum leaf proteomics. (A) Venn diagram of the overall proteins identified by each processing method. (B) A Pearson's correlation analysis between replicates and treatments. (C) Comparison of the digestion efficiency of OFIC, SDS, and TFA methods as represented by the percentage of peptides with 0, 1, or 2 missed cleavages. (D) Dynamic range of sorghum leaf proteome. The 20 most and least abundant proteins (top 20 and bottom 20) are indicated on the plot. Inner panel depicts the chloroplast proteins and their relative abundance. Numbers in the brackets indicate the number of proteins of each category.

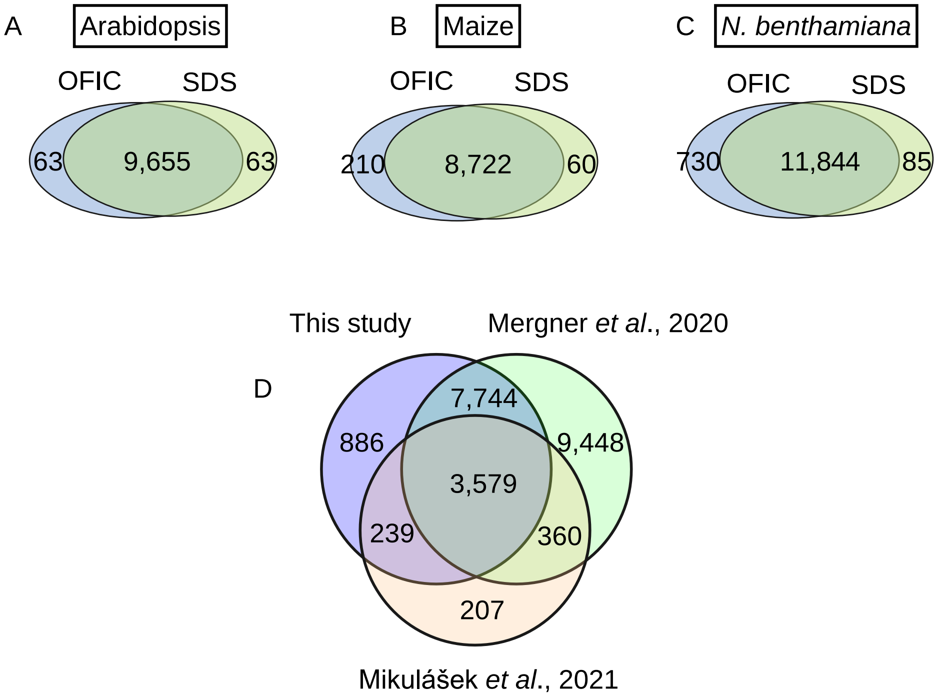

Supplementary Figure S3. Venn diagram analysis. (A-C) Comparisons of protein identifications derived from OFIC and SDS digestion methods for Arabidopsis, maize, and N. benthamiana leaves, respectively. (D) Overlapping analysis of Arabidopsis proteins identified by this study (in-cell digestion and single-shot LCMS) with two other studies by Mergner et al., 2020 (homogenization and urea-based in-solution digestion, high pH fractionation, and multi-shot LCMS), and by Mikulášek et al. 2021 (SDS lysate and single-shot LCMS). Please be noted that the numbers used here are slightly different from the numbers reported in the main text in this study and the Mergner's. We used all the protein accessions within each protein groups, if there are multiple members in one group, to maximize the overlaps between different studies.

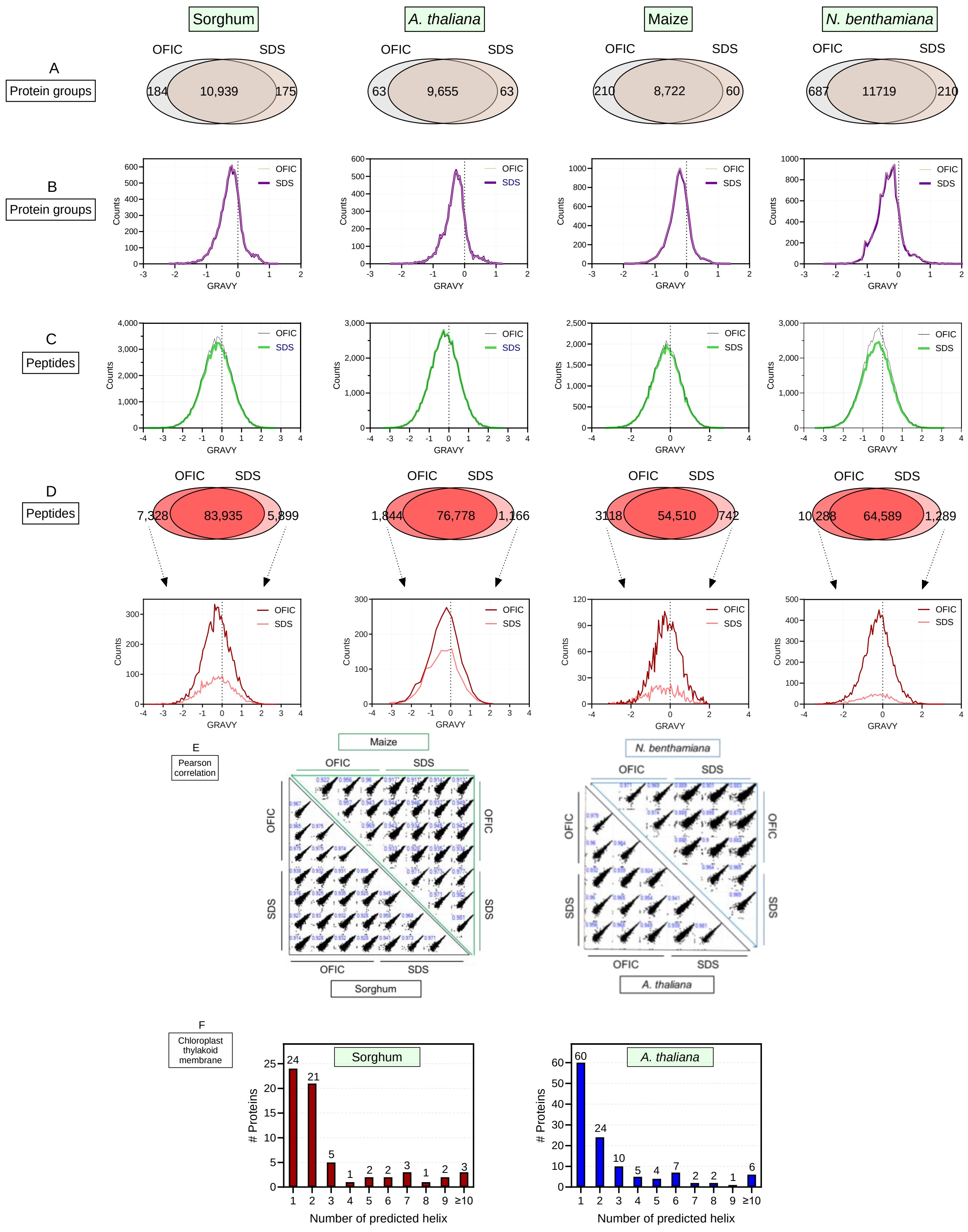
Sorghum
A. thaliana
Maize
N. benthamiana
A
Protein groups
OFIC
SDS
184
10,939
175
63
9,655
63
210
8,722
60
687
11719
210
B
Protein groups
Counts
GRAVY
C
Peptides
D
Peptides
7,328
83,935
5,899
1,844
76,778
1,166
3118
54,510
742
10,288
64,589
1,289
E
Pearson correlation
F
Chloroplast thylakoid membrane
Proteins
Number of predicted helix

Figure S4. **Overlapping, hydrophobicity and transmembrane analyses of OFC and SDS-base processing methods for leaf proteomics analysis**. (A) Venn diagram analysis of protein groups derived from the two processing methods. (B) Hydrophobicity profiles of protein groups obtained by the two methods. GRAVY scores were predicted using the GRAVY CALCULATOR online tool. (C) Hydrophobicity profiles of peptides obtained by the two methods. (D) Overlapping analysis of peptides obtained by the two methods. The lower panel shows the hydrophobicity profiles of the unique peptides detected in each method. (E) Pearson correlation analysis of the two methods. Four replicate experiments were included of sorghum and maize leaves (n = 4), and three replicates were included for *A. thaliana* and *N. benthamiana* leaves (n = 3). The correlation R values (highlighted in blue) were depicted in the plots. (F) Histograms showed the number of proteins that contained one or more transmembrane helix predicted by TMHMM predictor 2.0. The chloroplast thylakoid membrane proteins of sorghum and A. thaliana identified by OFIC method were plotted as representatives.

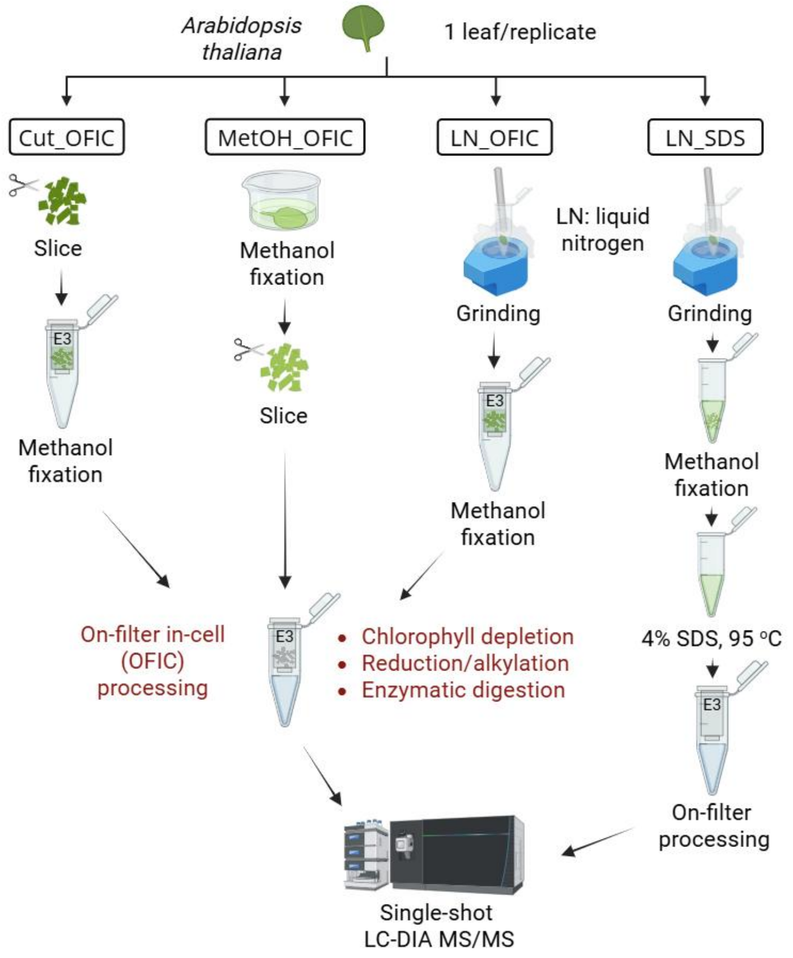

Supplementary Figure S5. **Illustrative workflow of the proteomic comparison of OFIC with grinding-based strategies.** *Arabidopsis thaliana* leaves were used for this study, and four different processing methods were compared. Three replicates (one leaf per replicate) were prepared by each method. In Method I (cut_OFIC), the rosette leaf was first sliced into small sections (1-2 mm^2) sections within a couple of minutes on ice, then transferred to E3filters and subjected to on-filter in-cell (OFIC) processing, which included pigment depletion, methanol fixation, reduction/alkylation, and protein digestion. In Method II (MetOH_OFIC), the leaf was first incubated with ice-cold methanol for around 5 min, and then sliced into small pieces followed similarly by the OFIC processing. In Method III (LN_OFIC), the leaf was first ground in liquid nitrogen, and then transferred to the E3filter and processed similarly according to the OFIC procedures. In Method IV (LN_SDS), after grinding in liquid nitrogen, the leaf powers were fixed with methanol, and then lysed with strong SDS buffer (4% SDS, 100mM Tris-HCl, pH 8) followed by on-filter processing using the standard E3 protocol. Three biological replicates (1 leaf per replicate) were performed in parallel for each method. The digests were submitted to single-run LC-MS/MS analysis using the same instrument configuration as described in the main text.

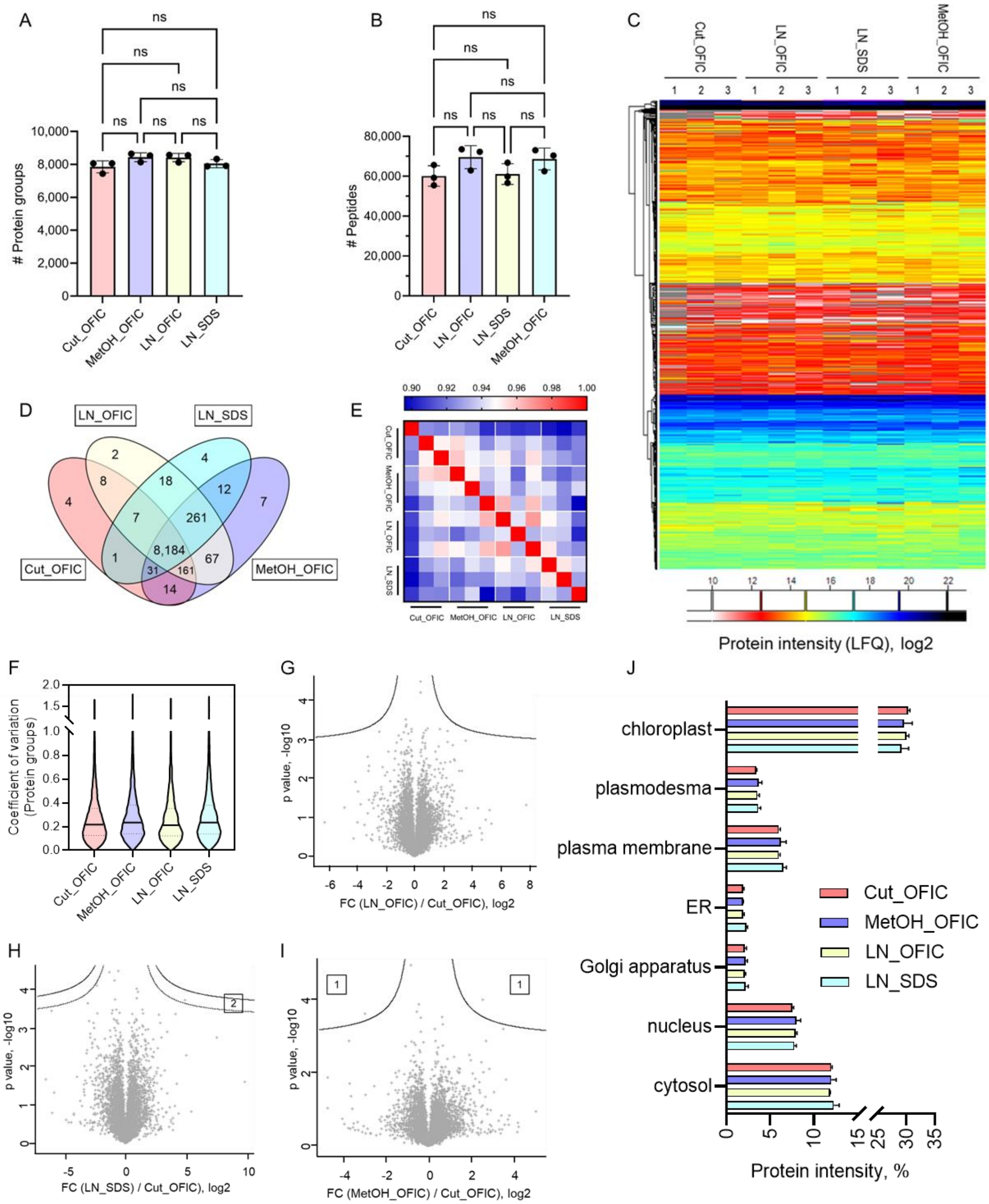

A
B
C
D
E
F
G
H
I
J
Protein groups
Peptides
Cut_OFIC
MetOH_OFIC
LN_OFIC
LN_SDS
ns
Protein intensity (LFQ), log2
Coefficient of variation (Protein groups)
p value, -log10
FC (LN_OFIC) / Cut_OFIC), log2
FC (LN_SDS) / Cut_OFIC), log2
FC (MetOH_OFIC) / Cut_OFIC), log2
chloroplast
plasmodesma
plasma membrane
ER
Golgi apparatus
nucleus
cytosol
Protein intensity, %

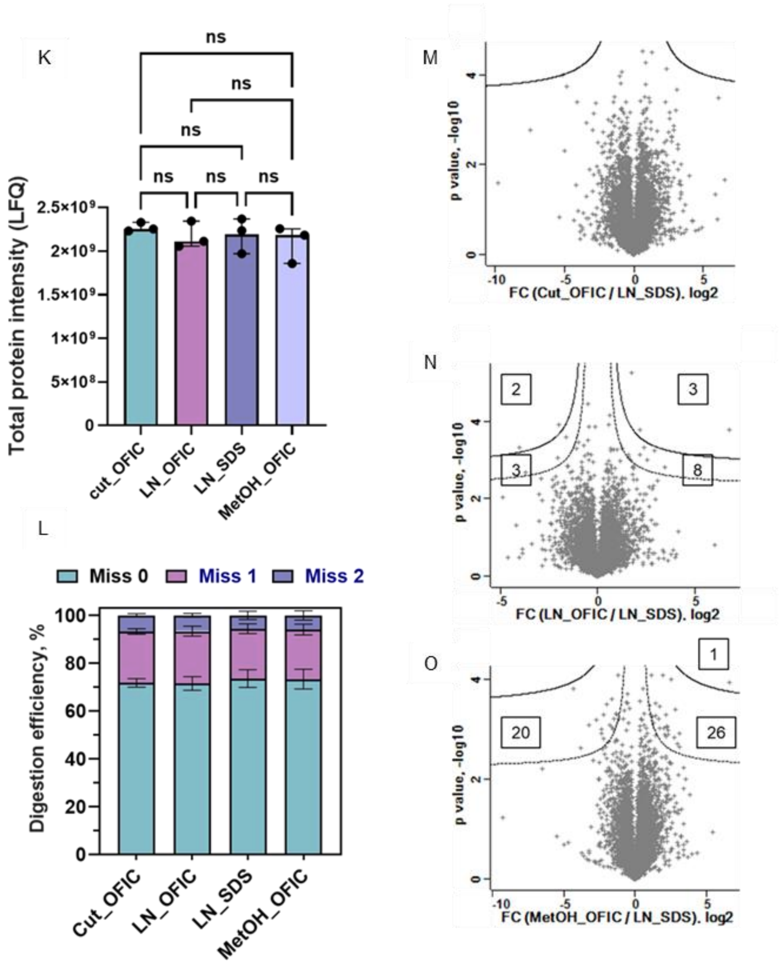

Supplementary Figure S6. **Quantitative assessment of different processing methods for *Arabidopsis thaliana* leaf proteomics.** (A-B) Numbers of protein and peptide identifications derived from the four processing methods as illustrated in **Fig. S4**. Error bars indicated three biological replicates (n = 3). (C) Coefficient of variation of protein groups derived from the four methods. Solid lines (black) indicate the median values. (D) Spearman rank correlation analysis of all the replicate experiments of the four methods. (E) Venn diagram shows the overlaps of proteins derived from the four methods. (F) Heatmap analysis of the protein groups obtained by the four processing methods. Protein intensity values were log2-transformed and color coded. Gray color indicates missing values. (G-I) Pair-wise t-test analysis of different processing methods. The method I (Cut_OFIC) was compared with the other three methods. The curves indicate S0 0.01 and FDR 0.05 (and FDR 0.01, if there are two curves). The boxed numbers show the number of significant proteins passed the two cutoffs. (J) Cellular compartment analysis of the proteins derived from the four processing methods. (K) Summed intensities of all the proteins quantified by each one of the four processing methods. (L) Percentage of missed cleavages. The intensity of all the precursors carrying 0, 1, or 2 miscleavages were summed and divided by the total precursor intensity. Error bars indicate three replicate experiments (n = 3). (M-O) Pair-wise t-test analysis comparing the LN_SDS method with the other three methods. The curves indicate S0 0.01 and FDR 0.05 (and FDR 0.01, if there are two curves). The boxed numbers show the number of significant proteins that passed the cutoffs.

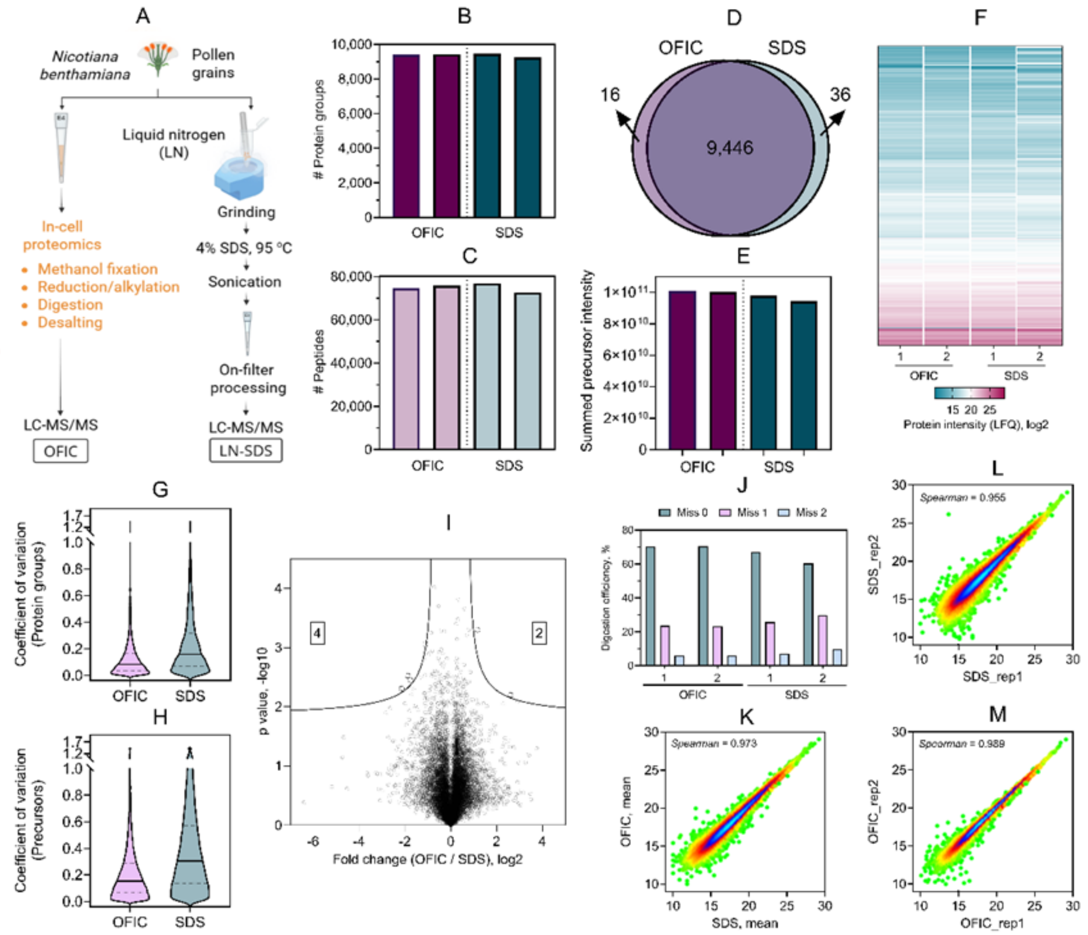

Figure S7. **Quantitative assessment of different processing methods for *Nicotiana benthamiana* pollen proteomics.** (A) Overview of N. benthamiana pollen proteomics workflow. In Method I (OFIC), the pollen grains were transferred directly to E4tips that were prefilled with methanol followed by OFIC processing, including methanol fixation, reduction/alkylation, digestion, and desalting. In Method II (SDS), the pollen grains were first ground in liquid nitrogen, then mixed with SDS buffer and boiled at 95 °C for 10 min. The lysate was further sonicated with water bath for 10 min, and then transferred to E3filters that were prefilled with 4x volume of 80% acetonitrile. The samples were then processed following the standard E3 procedures. The digests were desalted using C18 StageTip, dried, and analyzed using single-shot LC-MS/MS as described in the manuscript. Two biological replicates were prepared for each processing method. (B-C) Number of protein and peptide identifications obtained by the two methods. (D) Overlap of protein identifications between from the two methods. (E) Plot of total precursor intensity. (F) Heatmap analysis of proteins derived from the two methods. (G-H) Coefficient of variation of protein groups and precursors. Solid line (black) shows the median value. (I) Volcano plot analysis of the two methods. T-test fold change (log2) and p values (-log10) were plotted. Curves show the FDR cutoff 0.05 and S0 0.01. Boxed numbers show the number of significant proteins. (J) Plot of digestion efficiency. Intensities of precursors carrying 0, 1 or 2 missed cleavage sites were summed, and derived by the total precursor intensity. (K-M) Spearman's rank correlation analysis between the two methods (by mean values), or between the two replicates within the two methods.

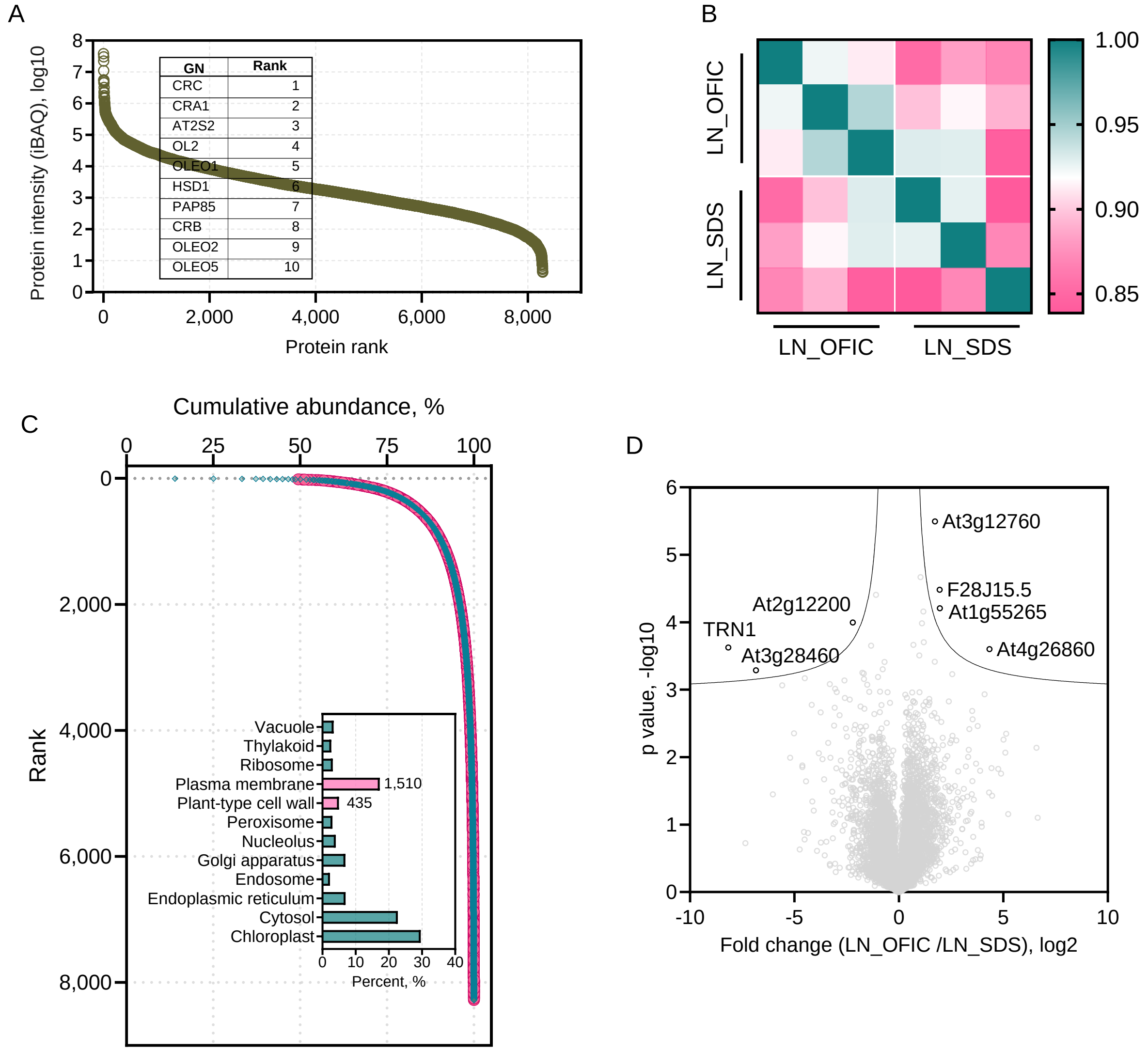

Figure S8. **Quantitative analysis of *A. thaliana* seed proteome.** (A) Proteome dynamic range profile. Inner table lists the top 10 most abundant seed proteins. (B) Correlation analysis. The Pearson r values are color coded. (C) Cumulative abundance plot. The inner bar diagram shows the cellular compartment distribution of OFIC-derived seed proteome. (D) Volcano plot analysis of the proteomes obtained by OFIC digestion and SDS-lysate based digestion.

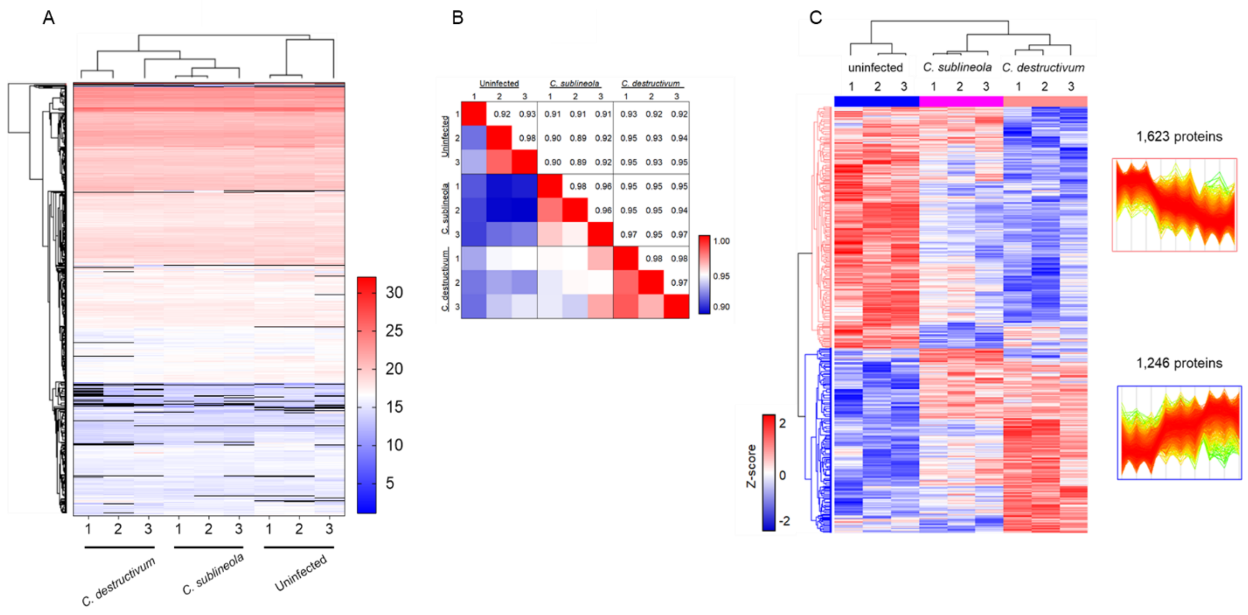

Supplementary Figure S9. Quantitative analyses of plant leaf infection. (A) Unsupervised hierarchical clustering analysis of the three groups. Dark lines indicate the proteins that have missing values. (B) Heatmap of Pearson correlation. (C) Heatmap of ANOVA significant proteins among the three groups. Significance cutoff is $p < 0.05$. The number of proteins in the two clusters (pink and blue highlighted) are depicted.

In-cell proteomics analysis of plant leaf using E3filter

1. **Sample pretreatment and loading**
   Collect leaves from plants; a 1-2 cm^2 section would be sufficient for one digestion experiment. Further slice it into 1-2 mm^2 pieces, and transfer them to E3filter.
   Note: The leaf collection and slicing should be completed within five minutes under low temperature (or on ice) to prevent potential stress response. Alternatively, the fresh leaves could be snap frozen with liquid nitrogen, and ground into small pieces (for instance, using pellet pestle with a cordless motor; Fisher Scientific; cat #: 12-141-361) before transferring to E3filters. Fine powder is NOT required at this step.

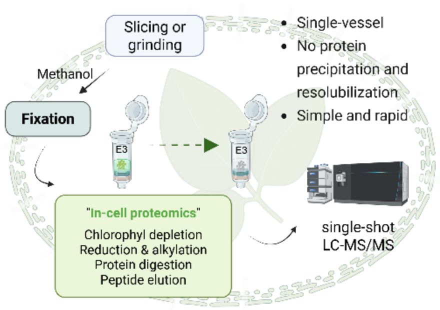

2. **Methanol fixation and chlorophyl depletion**
   Add appropriate amount of pure methanol (e.g., 100-400 µl) cover the leaves, shake at 1,000 rpm for 5 min under room temperature. Centrifuge at 3,000 rpm for 1 min, discard flow through. Repeat this step (e.g.,3-6 times) until the flow through is clear.
3. **Reduction and alkylation**
   Depending on the leaf size, add 100-300 µl of 50 mM triethylammonium bicarbonate (TEAB) buffer to cover the leaf pieces, and final concentration of 10 mM Tris(2-carboxyethyl)phosphine (TCEP), incubate at 45°C for 10-15 min with gentle shaking (300-500 rpm).
4. **Wash**
   Centrifuge at 3,000 rpm for 1 min to eliminate liquid. Add 200 µl of 50 mM TEAB solution, centrifuge again, and discard flow through.
5. **Digestion**
   Transfer E3filters to clean collection tubes (here, 2-ml tube is suggested), add 150 µl of 50 mM TEAB, desired enzyme (Trypsin or Trypsin/Lys-C mix) at 1:20 to 1:50 ratio. Incubate at 37°C for 16-18 hours with gentle shaking. For 1-cm^2, 1 µg of enzyme is suggested.
6. **Elution**
   After digestion, spin filters at 3,000 rpm for 1 min to collect flow through. Do two additional elution by adding 150 µl of 0.2% formic acid in water, and 150 µl of 0.2% formic acid in 50% acetonitrile, respectively, spin and collect flow through to the same collection tube.
7. **Drying and desalting**
   Dry digests in SpeedVac; do desalting separately following standard StageTip protocol.

In-cell proteomics analysis of plant pollen using E4tip

1. **Sample collection and loading**
   Collect pollen grains (e.g., with cold phosphate buffer), aliquot appropriate amount (e.g., 20-50 µl) to E4tips that are prefilled with 150-200 µl of pure methanol.
2. **Fixation**
   Spin tips at 4,000 rpm for 2 min, discard flow through. Add 200 µl methanol to samples, and incubate at room temperature for 15 min. Centrifuge at 4,000 rpm for 2 min, discard flow through. Tip: the flow through may be collected here for metabolomics analysis.

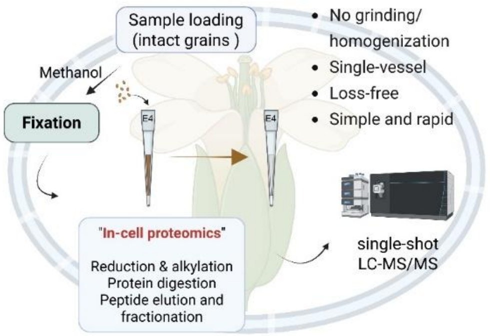

3. **Reduction and alkylation**
   Add final concentration of 10 mM Tris(2-carboxyethyl)phosphine (TCEP) and 40mM chloroacetamide (CAA) in 100 µl of 50 mM triethylammonium bicarbonate (TEAB), incubate at 45°C for 10-15 min with gentle shaking (300-500 rpm).
4. **Wash**
   Centrifuge at 4,000 rpm for 1 min to eliminate liquid. Add 200 µl of 50 mM TEAB solution, centrifuge again, and discard flow through.
5. **Digestion**
   Transfer E4tips to clean collection tubes, add 150 µl 50 mM TEAB, desired enzyme (Trypsin or Trypsin/Lys-C mix) at 1:50 ratio. Incubate at 37°C for 16-18 hours with gentle shaking.
   Tip 1: please make sure no air gap between the buffer and the membrane filter. Do a flash spin if desired (i.e., 2,000 rpm for 1 or 2 seconds).
   Tip2: No caps are required for E4tips during overnight incubation.
6. **Acidification and desalting**
   After digestion, add formic acid to final concentration of 1%, centrifuge at 1,500 rpm for 10 min. Add 200 µl 0.5% acetic acid in water, centrifuge at 4,000 rpm for 2 min, discard flow through.
7. **Elution**
   Transfer E4tips to clean collection tubes, do two sequential elution by adding 200 µl 60% acetonitrile/0.5% acetic acid in water (elution I), and 80% acetonitrile/0.5% acetic acid in water (elution II); centrifuge at 4,000 rpm for 2 min to collect eluants to the same tube. Dry samples in the SpeedVac, and store at -80°C. The peptides are now desalted and ready for LCMS analysis.

In-cell proteomics analysis of plant seeds using E3tip

1. **Sample collection and grinding**
   Collect mature plant seeds (e.g., with cold phosphate buffer), wash a few times, and grind in liquid nitrogen.
2. **Fixation**
   Add 200 µl methanol, shake at 1,000 rpm under room temperature for 5-10 min. Spin to discard supernatant. Repeat this step three times.
3. **Reduction and alkylation**
   Transfer the ground materials to E3tips, add final concentration of 10 mM Tris(2-carboxyethyl)phosphine (TCEP) and 40mM chloroacetamide (CAA) in 100 µl of 50 mM triethylammonium bicarbonate (TEAB), incubate at 45°C for 10-15 min with gentle shaking (300-500 rpm).

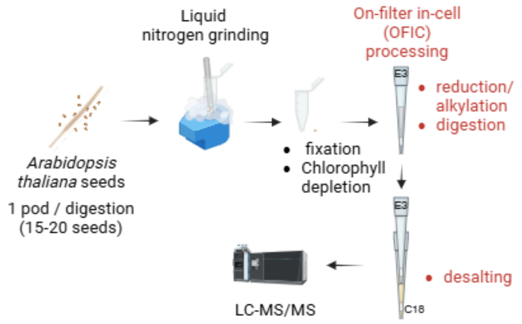

4. **Wash**
   Centrifuge at 4,000 rpm for 1 min to eliminate liquid. Add 200 µl of 50 mM TEAB solution, centrifuge again, and discard flow through.
5. **Digestion**
   Transfer E3tips to clean collection tubes, add 150 µl 50 mM TEAB, desired enzyme (Trypsin or Trypsin/Lys-C mix) at 1:50 ratio. Incubate at 37°C for 16-18 hours with gentle shaking.
   Tip 1: please make sure no air gap between the buffer and the membrane filter. Do a pulse spin if desired (i.e., 2,000 rpm for 2 seconds).
   Tip2: No caps are required for E4tips during overnight incubation.
   Tip3: For 15-20 *A. thaliana* seeds, 2 µg of trypsin is suggested.
6. **Acidification and desalting**
   After digestion, add formic acid to final concentration of 1%, incubate on bench for 3-5 min. Stack E3tips to pre-activated and pre-equilibrated C18 tips, centrifuge at 1,500 rpm for 10-15 min. Add 200 µl 0.5% acetic acid in water, centrifuge at 4,000 rpm for 2 min, discard flow through.
7. **Elution**
   Transfer the stacked tips to clean collection tubes, do two sequential elution by adding 200 µl 60% acetonitrile/0.5% acetic acid in water (elution I), and 80% acetonitrile/0.5% acetic acid in water (elution II); centrifuge at 4,000 rpm for 2 min to collect eluants to the same tube. Dry samples in the SpeedVac, and store at -80°C. The peptides are now desalted and ready for LCMS analysis.
